# A systematic review of normal tissue neurovascular unit damage following brain irradiation—Factors affecting damage severity and timing of effects

**DOI:** 10.1093/noajnl/vdae098

**Published:** 2024-06-13

**Authors:** Annet Nakkazi, Duncan Forster, Gillian A Whitfield, Douglas P Dyer, Ben R Dickie

**Affiliations:** Geoffrey Jefferson Brain Research Centre, Manchester Academic Health Science Centre, Northern Care Alliance NHS Group, The University of Manchester, Manchester, UK; Faculty of Biology, Medicine, and Health, Division of Informatics, Imaging, and Data Sciences, School of Health Sciences, The University of Manchester, Manchester, UK; Faculty of Biology, Medicine, and Health, Division of Informatics, Imaging, and Data Sciences, School of Health Sciences, The University of Manchester, Manchester, UK; Division of Cancer Sciences, Manchester Cancer Research Centre, Manchester Academic Health Science Centre, The University of Manchester, Manchester, UK; The Christie NHS Foundation Trust, Manchester, UK; Wellcome Centre for Cell-Matrix Research, Lydia Becker Institute of Immunology and Inflammation, Faculty of Biology, Medicine, and Health, Manchester Academic Health Science Centre, The University of Manchester, Manchester, UK; Geoffrey Jefferson Brain Research Centre, Manchester Academic Health Science Centre, Northern Care Alliance NHS Group, The University of Manchester, Manchester, UK; Geoffrey Jefferson Brain Research Centre, Manchester Academic Health Science Centre, Northern Care Alliance NHS Group, The University of Manchester, Manchester, UK; Faculty of Biology, Medicine, and Health, Division of Informatics, Imaging, and Data Sciences, School of Health Sciences, The University of Manchester, Manchester, UK

**Keywords:** brain irradiation, blood-brain barrier damage, cognitive decline, neurotoxicity, neurovascular unit dysfunction

## Abstract

**Background:**

Radiotherapy is key in the treatment of primary and secondary brain tumors. However, normal tissue is inevitably irradiated, causing toxicity and contributing to cognitive dysfunction. The relative importance of vascular damage to cognitive decline is poorly understood. Here, we systematically review the evidence for radiation-induced damage to the entire neurovascular unit (NVU), particularly focusing on establishing the factors that influence damage severity, and timing and duration of vascular effects relative to effects on neural tissue.

**Methods:**

Using PubMed and Web of Science, we searched preclinical and clinical literature published between January 1, 1970 and December 1, 2022 and evaluated factors influencing NVU damage severity and timing of NVU effects resulting from ionizing radiation.

**Results:**

Seventy-two rodents, 4 canines, 1 rabbit, and 5 human studies met inclusion criteria. Radiation increased blood-brain barrier (BBB) permeability, reduced endothelial cell number and extracellular matrix proteoglycans, reduced tight junction proteins, upregulated cellular adhesion molecule expression, reduced activity of glucose and BBB efflux transporters and activated glial cells. In the brain parenchyma, increased metalloproteinases 2 and 9 levels, demyelination, cell death, and inhibited differentiation were observed. Effects on the vasculature and neural compartment were observed across acute, delayed, and late timepoints, and damage extent was higher with low linear energy transfer radiation, higher doses, lower dose rates, broader beams, and in the presence of a tumor.

**Conclusions:**

Irradiation of normal brain tissue leads to widespread and varied impacts on the NVU. Data indicate that vascular damage is in most cases an early effect that does not quickly resolve. More studies are needed to confirm sequence of damages, and mechanisms that lead to cognitive dysfunction.

**Key Points**

- Brain irradiation induces widespread effects on neurovascular unit components.
- Damage extent is more severe with low linear energy transfer radiation, higher doses, lower dose rates, with broader beams, and in the presence of a tumor.
- Effects on the vasculature and neural compartment were observed at acute, delayed, and late timepoints.

**Importance of the Study**

The aim of radiotherapy is to deliver maximum dose to the tumor, while minimizing dose to surrounding normal tissues. For brain tumor patients, it is inevitable that healthy brain tissue is irradiated, leading to undesirable normal tissue toxicities. For patients that survive ≥6 months after brain irradiation, around 50% to 90% experience cognitive impairments, which significantly worsen life quality.^1^ Brain irradiation damages the vasculature and its coupling with brain cells,^2,3^ but factors that influence the severity of these changes, and their timings are not well understood. This systematic review identifies that neurovascular unit damage is worse with low linear energy transfer radiation, higher doses, lower (conventional) dose rates, broader beams, and in the presence of a tumor. A key result supports that both vascular and neural tissue changes occur at acute, delayed, and late timepoints. These findings should be taken forward to fully investigate when and how vascular changes may influence cognitive decline.

Radiotherapy (RT) is administered to approximately 60% to 70% of cancer patients globally as part of their curative or palliative treatment.^4,5^ For brain tumor patients that survive 6 months or more post-RT, around 50% to 90% experience some degree of cognitive impairments, such as loss/reduction in short-term memory, processing speed, executive function, and verbal fluency.^1,6^ While postirradiation cognitive impairment is a long-established clinical side effect,^7^ the biological mechanisms that lead to cognitive impairment are poorly understood.

Treatment for glioma includes safe maximal resection where possible, and/or RT (up to a total dose of 50 to 60 Gy, usually given in daily fractions of 1.8 to 2 Gy, 5 days a week^8–11^) and/or chemotherapy to improve local tumor control.^8,12–14^ The treatment combination chosen depends on factors including tumor histology, tumor location (particularly whether amenable to gross total resection), and patient age.^4,8,10^ Neurotoxicities arising from treatment are usually categorized into acute (arising during or up to 1-month post-RT), delayed (1 to 6 months post-RT), and late (≥6 months) effects.^15^ However, these timelines do not seem to be absolute, since various side effects can occur throughout acute and delayed phases,^16^ depending on factors such as patient characteristics, tumor type, and radiation modalities. Acute effects include nausea, vomiting, and headache, and are often related to edema,^15^ and usually resolve. Delayed effects include somnolence, transient demyelination, short-term memory loss, and occasional vomiting and nausea.^17–19^ Late complications include vascular abnormalities, tissue necrosis, and reduced cognitive abilities, which are usually progressive and irreversible.^15,19^ In preclinical studies, acute effects are generally considered to occur within 1-month, early delayed occur within 1 to 3 months, and late-delayed effects appear ≥3 months postirradiation,^20,21^ but the resolution rates can be shifted depending on factors like the species, strains, and radiation characteristics.^22^ Many patients will not live long enough to experience late irreversible cognitive impairment; however, for children or adult patients with curable or low-grade gliomas, these toxicities are a serious clinical issue.

Cognitive function relies on central nervous system homeostasis, which is maintained by 3 brain barriers: *the choroid plexus epithelium, arachnoid epithelium,* and *the* blood-brain barrier *(BBB)*^23^ (Supplementary Figure 1). These barriers modulate solute and fluid exchange between blood and cerebrospinal fluid or the brain, but the BBB exerts the greatest control over the brain microenvironment.^24,25^ Another key factor in maintaining healthy brain function is effective communication between the vasculature and neural compartment^26^ (Figure 1). This communication link, referred to as neurovascular unit (NVU), enables proper regulation of nutrient supply (to the brain tissue) and waste removal (from the brain tissue) at the right time, location, and amount^27^ (Figure 2). Any damage to the vasculature could impair the function of the BBB and NVU, potentially interfering with nutrient exchange and neurovascular coupling.

**Figure 1.**
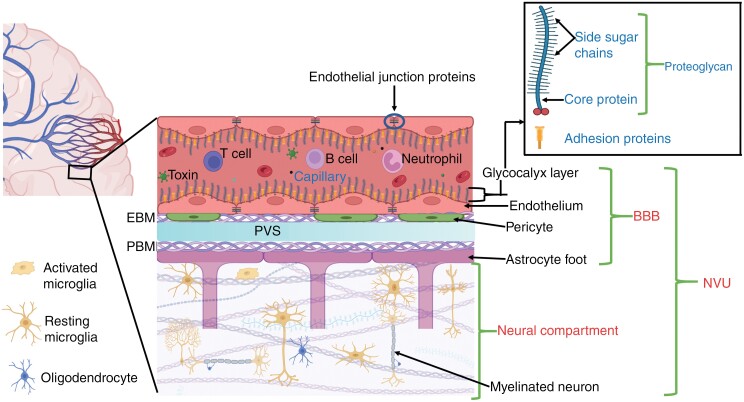
Components of the neurovascular unit in a healthy brain tissue. The BBB is the vessel component of the NVU, and is composed of the endothelial layer (endothelium and its glycocalyx), pericytes, endothelial basement membrane (EBM), perivascular space (PVS), parenchymal basement membrane (PBM), and astrocyte endfeet.^27,149^ Among these structures, the endothelial layer is considered to be the chief element of the BBB as it forms walls of the vessels, and regulates majority of the exchange/transport between blood and the brain tissue^150,151^ (Figure 2). The neural compartment consists of the interstitial matrix and perineuronal network,^73^ neurons, interneurons, oligodendrocytes, and resident immune cells (mainly microglia), which work closely with the BBB to meet the structural, developmental, and functional demands of the brain.^27,152,153^ This synergistic interconnection of NVU components enables proper neuronal metabolic activity, effective waste removal, a sufficient and well-regulated cerebral blood flow, and a controlled neuroimmune response.^154,155^ If the NVU is impaired, for instance by ionising radiation (IR), its ability to meet the energy demands of the neuronal tissue may be hindered, which could ultimately result in loss of proper brain function. (Figure created with BioRender.)

**Figure 2.**
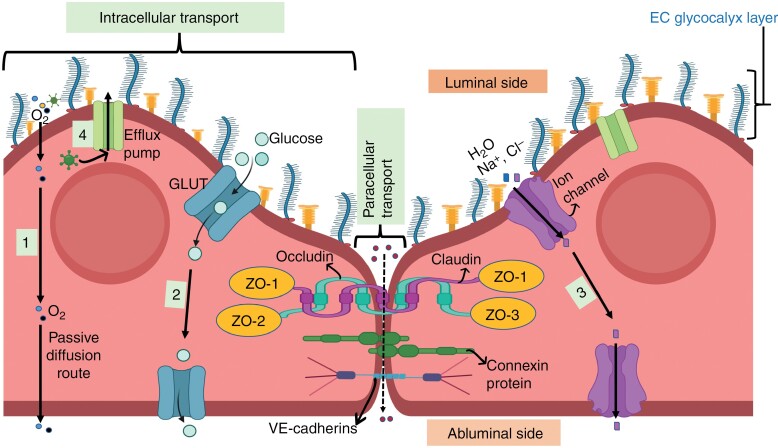
Endothelial layer components, and their role in regulating transport across the BBB. The endothelial layer is polarized into luminal (blood-facing) and abluminal (brain-facing) plasma membrane domains, and is made up of ECs (or endothelium) with their outer glycocalyx layer. Cerebrovascular ECs lack fenestrations,^156–158^ thus nutrients and other molecules enter the brain tissue via transcellular (through ECs) and/or paracellular (in between ECs) pathways,^159^ depending on factors like their molecular weight, lipid solubility, and charge.^160^ The transcellular pathway acts as the major transport route, and it allows crossing of small hydrophilic and lipophilic molecules (molecular weight <500 Da) through ECs by diffusion or specific transport channels.^161,162^ Small lipophilic molecules, such as oxygen, carbon dioxide, and alcohol can passively cross the endothelial cell membranes unrestricted (1), but glucose, and water and small ion molecules require specific transporters (eg, GLUTs) (2) and ion channels (3), respectively.^163^ To control passive diffusion into the brain, ECs highly express efflux pumps/transporters (4), mainly P-glycoprotein (a glycosylated member of the ATP-binding cassette transporters^164^) on the luminal side, which transport undesirable molecules like toxins back into the blood stream.^165,166^ Paracellular transport, on the other hand, is restricted by the 3 types of junctions that interconnect ECs; tight junctions (made of proteins, including zonula occludens—ZO, Occludin, and claudin),^167^ gap junctions (made of connexin proteins),^168^ and adherens junctions (made of proteins, such as VE-cadherins).^169^ These junction proteins only allow lipophilic, low molecular weight molecules to passively diffuse through the intercellular gaps, depending on hydrostatic, electrochemical, and osmotic gradient.^25,160,164,170–172^ This restriction is achieved due to their complex and layered arrangement, which creates a trans-endothelial electrical resistance (TEER) of up to 5900 Ω cm2 (in vivo, rat),^173^ making the BBB the tightest barrier in the body when compared to other organs’ TEER values that are below 4000 Ω cm2,^174,175,176,172^ However, the localization and expression of these junction proteins can be affected by stressors, such as upregulated Ca2+ signaling (due to increased intracellular Ca2+ levels) that has been reported to induce tight junction disassembly.^177,178^ Influx of molecules into the endothelium is also controlled by the glycocalyx (on the luminal side), a grass-like extracellular matrix (ECM) layer mainly composed of proteoglycans that mask cell adhesion molecules (CAMs).^179^ Proteoglycans consist of core proteins (mainly glypicans and syndecans) that are covalently bound to long unbranched glycosaminoglycan side chains (mostly chondroitin sulfate and heparan sulfate).^180^ CAMs include *selectins* (P and E-selectins, which are crucial in leukocyte adhesion) and *immunoglobulin-like proteins* (vascular cell adhesion molecule 1 [VCAM-1], intercellular adhesion molecules 1 and 2 [ICAM-1 and –2], and platelet/endothelial cell adhesion molecule 1 [PECAM-1, also known as CD31]).^181^ Immunoglobulin-like proteins are involved in cell-cell adhesion,^182^ EC migration and regulation of EC-matrix interactions, but their specific roles are not well established.^181^ If the endothelial layer is affected/damaged, toxic substances and peripheral immune cells can more easily enter the neural tissue, where they can induce unregulated immune responses and neuronal death. (Figure created with BioRender.)

The possibility that radiation damage to vasculature could contribute to cognitive dysfunction has led many groups to investigate the impact of radiation on the BBB or wider NVU.^20,21,28–32^ Recently, Allan and Limoli (2022) reviewed radiation-induced changes to the BBB and specifically focused on how these differ from changes to the brain tumor barrier. The review was not systematic in nature and did not capture when changes occurred. Hart et al. reviewed the effects of photon irradiation regimens on BBB permeability by systematically analyzing preclinical and clinical studies published before April 2020.^33^ Of the included clinical (*n* = 20) and preclinical (*n* = 49) studies, BBB disruption following RT was reported in 35% and 78% of the studies, respectively, and the severity was dependent on the protocol (ie, total dose, dose per fraction, and frequency) used. They found that in both clinical and preclinical studies, BBB permeability was increased at acute, delayed, and late-delayed timepoints, supporting the notion that BBB changes following brain RT are a chronic, not transient effect. While this was the first-time data from prior studies that had been synthesized to assess timing of vascular effects, the authors focused only on vascular permeability, considered only low linear energy transfer (LET) radiation and did not consider wider effects on the NVU. In this review, we aim to build on prior reviews by considering the impact of a wider range of ionizing radiation (IR) types (eg, photons vs protons) on both vascular and neural compartments, evaluating key factors affecting damage severity, and assessing the relative timing of vascular and neural compartment effects.

## Methodology

The aim of the review was to establish factors influencing severity of normal tissue NVU effects following IR, and to determine the timing (acute, delayed, and late) of such effects. Using the terms (Blood brain barrier AND (radiotherapy OR radiation OR irradiation) AND normal tissue) OR (Endothelial cells AND (radiotherapy OR radiation OR irradiation) AND normal tissue)) OR (Pericytes AND (radiotherapy OR radiation OR irradiation) AND normal brain tissue)) OR (Tight junctions AND (radiotherapy OR radiation OR irradiation) AND normal tissue)) OR (Astrocytes AND (radiotherapy OR radiation OR irradiation) AND normal brain tissue)) OR (Glycocalyx AND (radiotherapy OR radiation OR irradiation) AND normal brain tissue)) OR (Extracellular matrix AND (radiotherapy OR radiation OR irradiation) AND normal brain tissue)) OR (Neural tissue AND (radiotherapy OR radiation OR irradiation) AND normal tissue), we searched PubMed and Web of Science databases for literature published between January 1, 1970 and December 1, 2022. After a thorough scanning of titles and abstracts, a review was done in accordance with the Preferred Reporting Items for Systematic Reviews and Meta-Analyses (PRISMA) guidelines.^34^ Literature before 1970, letters, reviews, non-English articles, conference abstracts, non-IR studies, IR studies in non-brain tissues, studies on only tumor tissue, and other cancer treatments were excluded. Included studies were classified based on subject traits, IR characteristics, regions of focus/interest, and timelines (follow-up time), and then quantitatively analyzed. To note, the biological effective doses (BED), as one of the key IR characteristics, were calculated using the linear-quadratic equation by Fowler (1989),^35^ that is, BED = total dose [1+ dose per fraction/(α/β)], where α/β value of 3 was used for normal brain tissue.

## Results

**Figure 3.**
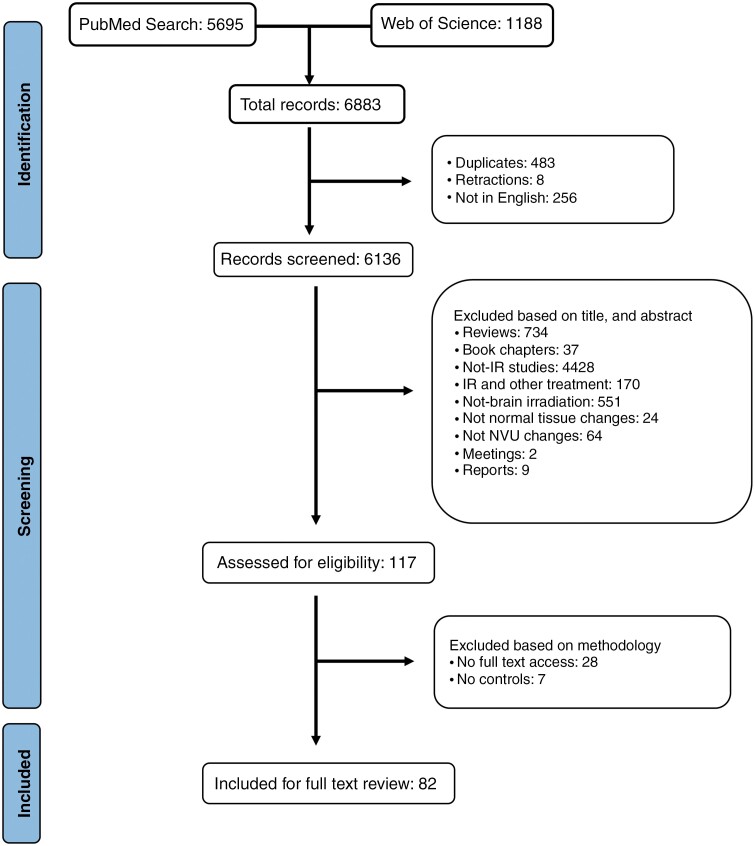
PRISMA flow diagram showing inclusion and exclusion criteria of identified publications.

The number of studies identified by the search terms and included within the systematic review are shown in Figure 3. A total of 6883 records were collected into EndNote (version 20) from PubMed and Web of Science. After removing copies, retractions, and non-English-language articles, a total of 6136 articles were screened.

**Figure 4.**
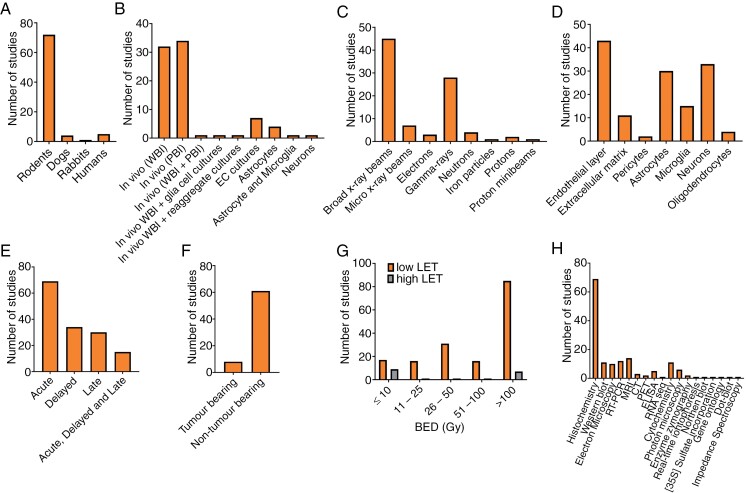
Summary of preclinical and clinical studies investigating radiation-induced effects on the NVU. A quantitative analysis of (A) subjects studied, (B) models, (C) ionizing radiation type, (D) NVU component studied, (E) timepoints studied, (F) studies with and without tumors present, (G) biologically effective doses (BED) used separated into high and low LET types, and (H) assays used.

Study characteristics are summarized in Figure 4. Seventy-two rodents, 4 canines, 1 rabbit, and 5 human studies met the inclusion criteria (Supplementary Tables 1 and 2). Of the 77 preclinical studies, 68 were in vivo, and only 7 used tumor-bearing models. Of the 5 clinical studies, only one was in vivo. Changes were mainly reported in the endothelium (*n* = 43), neurons (*n* = 33), astrocytes (*n* = 30), microglia (*n* = 15), and the extracellular matrix (ECM) (*n* = 11).

**Figure 5.**
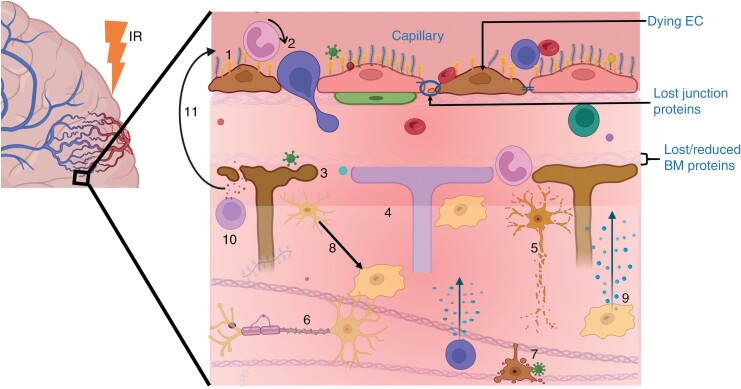
A summary of radiation-induced NVU damage/changes, and how the neural tissue can be directly or indirectly affected. Indirect effects can start from BBB disruption: Loss of the glycocalyx proteoglycans results in exposure of CAMs, such as selectins, ICAM-1, and VCAM-1 (1), which enable circulating immune cells like leukocytes to infiltrate through the endothelium (2). Additionally, loss of ECs, junction proteins, pericytes, and basement membrane (BM) components allows easier influx of blood components (like toxins and immune cells) into the brain tissue where they can be harmful to astrocytes (3) and neural tissue cells (7). Direct effects to the neural tissue can involve loss of neuroblasts, interstitial ECM proteins (4), neurons (5), myelin sheath (6), oligodendrocytes and microglia (7), reduced stem cell proliferation and differentiation, and dysfunction of synaptic and volume transmission. In both pathways, infiltrating and resident immune cells become activated (8), which triggers signal transduction pathways, such as the nuclear factor kappa B, that mediate the production of proinflammatory cytokines, chemokines and inducible enzymes (9 and 10).^183^ These mediators in turn increase the BBB permeability (11), for instance, by further upregulating the expression of adhesion molecules on the endothelium,^184^ and the cycle repeats. (Figure created with BioRender.)

The following sections summarize observed radiation-induced changes to NVU components (shown in Figure 5), factors influencing size of effects, and timings of vascular and neurocompartment effects in relation to acute, late, and delayed time intervals.

### Impacts of IR on NVU Components

#### Endothelial layer.—

The endothelial layer includes endothelial cells and junctional proteins that regulate endothelial permeability/transport (Figures 1 and 2), and surface proteins, mainly cellular adhesion molecules (CAMs), which play key roles in initiating inflammatory responses upon insult to the brain (Figure 2).

Of the 43 studies that examined endothelial layer changes, 19 showed an increased endothelial permeability to serum antibodies/proteins and injected tracers (such as Evans Blue),^20,22,30,32,36–49^ while 7 showed no change in permeability.^21,22,32,43,50–52^ Elevated ICAM-1,^41,42,52–58^ E-selectin,^58,59^ and VCAM-1^56^ expression levels, upregulated PAI-1 expression and intracellular reactive oxygen species (ROS),^57^ loss of tight junctions,^39,60,61^ a significantly reduced endothelial barrier tightness,^46^ EC loss,^51,56,62^ a 60% drop in the P-glycoprotein density,^63^ and increased Ca2+ signaling^64^ were also reported, which all promote endothelial hyperpermeability. Additional changes reported on the endothelium include; increased vesicular activities,^39^ enlarged/dilated vessels,^30,42,49,51,61,65–67^ swollen ECs,^50,68^ blood vessel wall separation from basal membrane (perivascular edema),^36^ a significantly increased average distance between Ki-67+ cells (marker for cell division) and vessels^69^ an increased cross-sectional area of the vessels,^20,31,49^ reduced vessel length density,^67^ disorganized vascular network in the ipsilateral normal tissues,^49,51,67^ blood vessel wall thickening,^36,60,70^ and a significantly reduced blood flow.^71^ A few studies that examined changes in expression of angiogenesis proteins reported a significantly increased expression of VEGF,^44,51,60,72^ chemokine (C-X-C motif) ligand 12 (CXCL12),^72^ and angiopoietins-2 (ANG-2, the ANG-1 inhibitor).^60^ Surprisingly, expression levels of ANG-1 and an endothelial-specific receptor tyrosine kinase (TIE-2, which promotes a well-regulated angiogenesis with ANG-2 binding) were greatly reduced.^60^

#### Extracellular matrix.—

The ECM mainly consists of a variety of proteins, glycans, and glycoproteins that not only provide structural and signaling support to the BBB (by the glycocalyx layer and vascular basement membranes) and to the neural compartment (by the interstitial matrix and perineuronal network),^73,74^ but are also crucial in guiding immune cell movement and positioning.^75^ Nonetheless, radiation-induced changes in these matrix components seem to be overlooked. Key reported changes in the ECM were reduced expression of glycosylated proteoglycans^76^; a significant loss of glycosaminoglycans (sugar chains on proteoglycans) with a 2–2.5-fold decrease in overall cortical content of heparan sulfate (*P* < .05)^77^; a thickened basement membrane in ipsilateral (irradiated) cortices^47,50^; cortical cytoskeletal component loss^36,51^; a significant upregulation of metalloproteinases (MMP)-9, MMP-2 and tissue inhibitor of metalloproteinase-1 (TIMP-1—the MMP-9 inhibitor) in the hippocampus and the cortex, an increase in gelatinolytic activity of MMPs, and a significant drop in hippocampal collagen type IV levels compared to controls^78^; and a significantly increased MMP-9 colocalization and correlation with CD147 (*R*^2^ = 0.834, *P* < .001).^45^

#### Pericytes.—

Pericytes are key in maintaining the endothelial structure^79^ and regulating cerebral blood flow.^80^ Of the 2 studies that examined effects in pericytes,^69,81^ only 1 reported changes in pericyte coverage (marked by CD13+ cells) after mice exposure to a single whole brain x-irradiation dose of 8 Gy.^69^ At 6 hours post-IR, they found a 17% decrease in CD13+ cells in the subgranular zone (SGZ), but no significant differences in the rest of the dentate gyrus (DG). At all the remaining time points (1 and 7 weeks), CD13+ cell numbers did not differ from those of controls.^69^

#### Glia.—

Astrocytes and microglia are key glial cells that support both neuronal function and BBB integrity. Astrocytes link blood vessels to neurons, and they are the main orchestrators of water transport into the brain and neurovascular coupling.^82^ Microglia, on the other hand, are the brain’s primary resident immune cells, which play central roles in brain immune responses and in BBB maintenance.^83,84^ Loss of astrocytes and ECM components weakens the endothelium, which ultimately provides a quicker access of blood components, such as immune cells, to the neural tissue where they can induce undesirable immune responses.^85,86^ Upon brain injury, astrocytes and microglia appear to coordinate and respond as one unit,^87^ thus, disruptions in their activities can significantly affect brain function.

Thirty studies examined changes in astrocytes. Increased production of plasminogen activators,^88^ loss of astrocytes in rat cortex^89^ and subependyma,^90^ and increased expression of DNA repair proteins^91^ were observed. Other glial cell studies reported a significant loss of hippocampal microglia,^92^ elevated reactivity of astrocytes and microglia in cortex and hippocampus of rabbits,^66^ dogs,^31,65^ and rodents,^20,22,36,37,39,41,44,49,50,52–54,93–95^ an increase in CD68+^96^ or EDI+^37^ (markers for mainly activated microglia/macrophages^97,98^) cells in rats, and a decreased glial cell viability and ability to restrict tumor cell progression.^77^ In contrast, a few studies showed neither damage to normal astrocytes^30^ nor gliosis.^21,95^

#### Neurons and oligodendrocytes.—

Neurons are the central elements of an entire cascade of signal transduction for proper cognition^99^ as they form the necessary circuits for coding, processing, and storage of information in the brain.^2^ Efficient signal transmissions within the brain require healthy neuronal structures (ie, synapses, soma, axons, and neuroeffector junctions). Oligodendrocytes produce myelin, a protein that insulates the axons, thus, these cells are essential in maintaining proper electrical signaling.^100^

A number of studies reported significant loss of mature neurons^37,42,89,90,101–108^ and dividing neurons^42,106,108^ in various brain regions, mainly in the DG and subventricular zone (SVZ). Loss of oligodendrocyte progenitor cells^103^ and oligodendrocytes^109^ was also found. Other findings include, inhibited neuronal maturation^21,94,108^; axonal swelling and inhibition of hippocampal neurogenesis,^42^ dose-dependent reduction (≥30%) in myelin synthesis^110^; myelin sheath swelling^36,42,66^; myelin loss/fragmentation^20,22,30,65,93,103,105,107,109,111,112^; an increased expression of neuron and specific enolase^65^; an increase in extracellular space volume fraction, tortuosity and nonspecific uptake^37^; tissue necrosis in the hippocampus,^22,40^ white matter,^31,37,65,70^ cortex,^20,37^ corpus callosum and septum^62^; cavitation and mineralisation^22^; mitochondrial alterations, an increased density in synaptic contacts, rarefaction of the presynaptic vesicles, and widened presynaptic pole,^36^ a dose-dependent increase in levels of ROS/RNS and nitric oxide,^108^ an increased γH2AX signal (marker for DNA double strand breaks), especially in the DG,^108,113^ overactivation of AKT and MAPK intracellular signaling pathways, and overexpression of several neurotrophins and their receptors,^114^ and chromatin condensation and aggregation in ipsilateral cortex.^47^

### Factors Modulating IR Effects on NVU Components

#### Dose effects.—

Ernst-Stecken et al. compared the impact of various fractionated x-ray doses (10 Gy/fraction/week) on immunoglobulin G (IgG, 150 kDa) leakage into the hippocampus. They found no changes in IgG at 20 Gy, but observed increased IgG at 12 weeks in the irradiated side with 30 Gy and at 8 weeks following 40 Gy.^40^

Serduc et al. examined effects of dose on leakage of 0.58 kDa sulforhodamine B (SRB) and 70 kDa fluorescein isothiocyanate (FITC)-dextran dyes. Microbeams with a single entrance dose of either 312 or 1000 Gy were applied to the cortex. At all time points and doses studied, the 70 kDa dextran dye remained in the vessels, while 0.58 kDa SRB leaked between 12 hours and 12 days for 1000 Gy, but not 312 Gy.^32^

Suckert et al. investigated dose effects on BBB leakage and edema formation using C3H/He (radiation sensitive^115^) and C57BL/6 (radio-resistant^116^) mice. They irradiated the right hippocampi with single-dose proton irradiation (40–45, 60–65, or 80–85 Gy) and scanned mice with DCE-MRI and T2-w MRI. They found leakage and hyperintense MRI signal occurred in the hippocampus and white matter of both strains at similar times.^49^ At doses below 40–45 Gy, T2-weighted MRI hyperintensities, indicative of edema, were not seen at any timepoint. At the higher doses, hyperintense signals were sustained up to 11–13 weeks, and followed the same spatial pattern as BBB changes, suggesting edema was caused by BBB breakdown.

Sharma et al. investigated dose effects (2, 5, or 10 Gy) of gamma rays on trans-endothelial electrical resistance (TEER; a measure of barrier integrity), and the localization of cytoskeleton proteins (F-actin), junction proteins (VE cadherin and ZO-1) and PECAM-1 of cultured human ECs.^46^ At 3 hours post-IR, they observed a dose-dependent significant decrease in TEER values and PECAM-1 levels, but no difference in localization of actin cytoskeleton, VE cadherin, and ZO-1. TEER values dropped by 100 Ω at 2 Gy, 200 Ω at 5 Gy, and 450 Ω at 10 Gy. However, by 6 hours post-IR, TEER values and PECAM-1 localization were similar to controls,^46^ indicating a rapid restoration of endothelial protein.

Ljubimova et al. investigated the impact of dose on EC populations. Whole rat brains were exposed to single x-ray doses between 2.5 and 200 Gy.^62^ At 2.5 Gy, no effects were observed. For doses greater than 2.5 Gy, reductions in EC number of at least 15% were observed in cortex, septal area, and corpus callosum, and lasted from 24 hours to 65 weeks. Similar results were found in mice ECs culture treated with single x-ray doses of 5, 15, or 25 Gy. Here, the authors observed a significantly reduced number of viable ECs, increased cellular hypertrophy and enlarged nuclei from 24 to 72 hours for 15 and 25 Gy,^56^ but not 5 Gy. Bouchet et al. reported similar EC loss in peritumoral normal tissues (ie, caudate nucleus) 45 days after a single microbeam radiotherapy (MRT) dose of 312 Gy,^51^ and in cortex of naive irradiated rats at 1-month post-a single gamma-ray dose of 60 Gy. In contrast, gamma knife surgery (GKS) was found to increase EC division 3–6 months following 60 Gy,^47^ 4–6 months following 75 Gy, and at 1 month following 120 Gy,^20^ suggesting higher doses may cause possible EC remodeling to occur sooner. Similarly, a study on cultured human brain ECs exposed to a single gamma-ray dose of 50 Gy showed a significantly increased lactate dehydrogenase (cell stress and necrosis marker^91^) activity at 72 hours post-IR, but no increase in caspase-3 activity up to 120 hours compared to nonirradiated controls.^58^ Some studies in naive mice found no effects up to 3 months using single MRT doses (312 or 1000 Gy)^32^ and up to 6 months following single dose (0.5–4 Gy)^56^Fe particle therapy (high LET IR).^67^

Olschowka et al. hemi-irradiated mice with single gamma-ray doses between 5 and 35 Gy, and observed increased ICAM-1 mRNA levels in ipsilateral hemispheres at 6 hours for dose >15 Gy. Additional histological examination of the 25 Gy group showed greatly increased ICAM-1 staining in hippocampi and parietal cortices of both hemispheres at all time points (4 hours to 7 days) compared to controls.^53^ Several other studies also found increased ICAM-1 expression at 24 hours post-35 Gy single-dose whole brain gamma-irradiation,^54^ between days 1 and 7 post-30 Gy single-dose partial brain x-irradiation,^55^ and between 4 and 24 hours following whole brain x-irradiation.^42^ Murine EC culture studies observed similar findings. A dose- and time-dependent increase in expression of ICAM-1 and VCAM-1 was found after single 5, 15, or 25 Gy doses of gamma-rays,^56^ a time- and dose-dependent increase in levels of ICAM-1 and PAI-1 expression and intracellular ROS up to 1 day post-single doses (1, 2, 5, or 10 Gy) of gamma rays (*P* < .05),^57^ and a short-term (up to 1 week) significant downregulation of E-selectin levels post-a single 25 Gy of x-rays.^59^ Interestingly, VCAM-1 and E-selectin levels in irradiated cultured human ECs were not different from those of controls despite a significant upregulation of ICAM-1 levels (*P* < .001) at 24 hours following 50 Gy dose of gamma rays.^58^

Chiang et al. studied the impact of gamma dose (2, 4, or 8 Gy) on cultured mice astrocytes and microglial cells and found no significant increases in tumor necrosis factor (TNF)-alpha (also key in immune responses) production compared to controls at any time point (4 to 24 hours) post-IR.^117^

In an ex vivo study, Acharya et al. explored dose effects by exposing human neural stem cells to 1, 2, or 5 Gy of gamma-irradiation and examined various cellular changes, including multipotency and differentiation (at 2 days post-IR), cell number and metabolic viability (at 3, 4, and 5 days post-IR), and oxidative stress (at 7 days post 2 or 5 Gy).^108^ Irradiated cells expressed markers associated with an undifferentiated state (nestin and Sox2), and formed significantly fewer immature neurons (B-III-tubulin+ cells) compared to controls. At all time points post-all doses, cell number and metabolic ability significantly decreased compared to those of controls. By 6 hours post-5 Gy, apoptosis in irradiated cells increased by over 2-fold, reached a peak (3-fold) by 12 hours, and then dropped to baseline levels by 48 hours post-IR. Compared to controls, levels of ROS/RNS and nitric oxide increased by 3-fold after 2 Gy. After 5 Gy, ROS/RNS levels increased by 7-fold and nitric oxide by 5-fold. Superoxide levels in both 2 and 5 Gy doses did not change significantly compared to control levels.^108^

A number of studies investigated dose effects on myelin loss. Janzer et al. permanently hemi-implanted dogs with segments of Ir-192 seed (gamma-emitter, 0.05 Gy/h), and histologically examined brain tissue changes between 25 and 362 days post-implantation.^65^ Throughout the study period, demyelination (in white matter) and an increased expression of neuron-specific enolase were evident in ipsilateral cortex and basal ganglia but not in control animals. A comparable dose-dependent myelin loss was also reported from a magnetic resonance imaging (MRI on a 9.4 T magnet) rat study, which was indicated by significantly reduced functional anisotropy and increased axial/radial diffusivity at 2 months post-single PBI doses (150–500 Gy) of micro- or minibeams compared to controls (*P* < .05).^112^

Taken together, these observations suggest that endothelial leakage/permeability, ICAM-1 and VCAM-1 upregulation, EC loss/death, EC division, ROS/RNS levels, and myelin loss are greater with larger doses. There is some evidence that larger doses cause changes to occur earlier in time.

#### Low LET versus high LET.—

Coderre et al. compared low and high LET radiation types. They hemi-irradiated tumor-bearing rats with a single 13.4 Gy dose of boronphenylalanine (BPA)-based boron neutron capture therapy (BNCT; high LET) or a single 22.5 Gy dose of x-rays (low LET). At 1-year post-BNCT, mild horseradish peroxidase (HRP, 44 kDa) leakage was found in the irradiated side, whereas in the x-ray-treated group, significant extravasation was found in both ipsilateral and contralateral tissue compared to both BNCT and control groups.^30^ The same study investigated effects of low LET versus high LET on neuronal microstructure. Minor atrophy of the corpus callosum was found at 1 year following BNCT, but severe cortical and white matter atrophy was evident following 22.5 Gy of x-irradiation,^30^ which occurred bilaterally. Conversely, 2 studies found no change in cortical neuronal structures of either hemi-irradiated rats at 3.5 months post-a single GKS dose of 75 Gy^50^ or wholly irradiated rat brains at 12 months post-a fractionated gamma-ray dose (both low LET) of 45 Gy (5 Gy per fraction, twice a week).^118^

These observations suggest that endothelial leakage to HRP and white matter demyelination is more severe (or more easily detectable) with low LET IR. Cortical neuronal structures appear less affected, and could be more robust to IR than white matter.

#### Dose rate.—

Allen et al. evaluated the impact of dose rate on endothelial junctional proteins, which have key roles in maintenance and function of a tight endothelial barrier. Whole mice brains were exposed to single electron-beam doses of 10 or 25 Gy at conventional (0.09 Gy/s), or ultra-high (ie, ≥40 Gy/s^119^) FLASH (5.6 × 10^6^ Gy/s for 10 Gy, 6.9 × 10^6^ Gy/s for 25 Gy) dose rates, and examined changes in tight junction protein expression and endothelial nitric oxide synthase (eNOS) activity at 24 hours and 1-week post-25 Gy or at 1-month post-10 Gy.^61^ At 24 hours post-25 Gy, FLASH IR induced a significant increase in hippocampal claudin 5 levels compared to conventional and sham control groups. At 1 week, claudin 5 and occludin levels in the hippocampus and SVZ significantly dropped in the conventional, but not in FLASH IR or control groups. At 1-month post-10 Gy, both dose rates induced a significant reduction in occludin expression, but a decrease in claudin 5 was only present in conventional cohorts. At 1-week post-25 Gy and 1-month post-10 Gy, the activity of eNOS significantly increased in the SVZ and DG of conventional dose-rate groups, but not in FLASH and control groups, highlighting that ultra-high dose rates may be less damaging to brain vessels.

Montay-Gruel et al. investigated the impact of dose rate on astrocyte reactivity. Mice whole brains were irradiated with single 10 Gy dose of electron beams at conventional (0.09 Gy/s) or FLASH (5.6 × 10^6^ Gy/s) IR dose rates. They revealed a significant increase in complement component 1q (C1q) expression, complement component 3 (C3) immunoreactivity and GFAP+/C3 co-labeling throughout the brain at 1-month post both dose rates. However, in conventional groups only, there was a significantly elevated IBA1+/C1q co-labeling, an increased toll-like receptor 4 (TLR4) expression, and a rise in TLR4+/GFAP+ cells in the hippocampus.^95^

In summary, the impacts of IR on junctional proteins, eNOS, IBA1+/C1q co-labeling, TLR4 expression, and TLR4+/GFAP+ cells were less with higher dose rates.

#### Single versus fractionated delivery.—

Lee et al. studied the effects of a single (10 Gy, in rats) versus fractionated (40 Gy; 5 Gy/fraction/day, twice a week, in mice) on the activity of MMPs (these cleave proteoglycans and degrade other ECM components^120^) at 4, 8, and 24 hours post-IR.^78^ A single 10 Gy dose caused significant upregulation of MMP-9 and MMP-2 mRNA and tissue inhibitor of metalloproteinase-1 (TIMP-1—the MMP-9 inhibitor) in the hippocampus and the cortex at all time points, but did not change levels of MMPs-3, 7, 10, 12, or TIMP-2 (the MMP-2 inhibitor). In the fractionated dose group, hippocampal MMP-2 mRNA was similarly upregulated at all time points, but again, TIMP-2 was not affected. Unfortunately, changes in other MMPs were not investigated in this group. In both single (rats) and fractionated (mice) groups, there was a significant increase in gelatinolytic activity of MMPs and a significant drop in collagen type IV levels in the hippocampus at 24 hours post-IR compared to controls.^78^ Likewise, when Li et al. hemi-irradiated rats with a single 75 Gy dose of GKS, they found significantly increased CD147 and MMP-9 protein expression levels only in the ipsilateral cortex from 8 to 12 weeks post-IR compared to no-IR controls.^45^ Double staining at 12 weeks post-IR showed MMP-9 colocalization and significant correlation with CD147 in the vascular lumen-like structure compared to controls.

In summary, both single and fractionated IR cause upregulation of MMP and TIMP-1, but there was insufficient data to draw conclusion about the impact of fractionation on severity of effects.

#### Broad versus focused beams.—

Prezado et al. irradiated whole rat brains with a single 25 Gy dose of protons delivered as broad beams (PRT) or minibeams (pMBRT) and examined changes in BBB leakage and myelin loss at 10, 90, and 180 days post-IR. They observed increased leakage of gadolinium-DOTA (Gd-DOTA, 0.56 kDa) and edema in the hippocampus, hypothalamus, ventricles, basal forebrain, brainstem, and septal regions for PRT group only, and only at the 180-day timepoint.^22^ Demyelination mainly in the hippocampus and hypothalamus was found at 6 months post-PRT, but not in pMBRT treated groups,^22^ indicating that delivery of radiation fields using focused beams may have less impact on vasculature and myelin than broad beams. On the other hand, when Bouchet et al. compared a 10 Gy dose of broad x-ray beams (BB) and a 241.4 Gy of MRT in rats, MRT induced a significant ipsilateral increase in Gd-chelate extravasation (up to 1 week) compared to BB-irradiated tissues (*P* = .0085) and controls (*P* = .0242).^48^ Since doses used here differ, more studies will be needed to make stronger conclusions.

#### Presence/absence of tumor.—

Zawaski et al. investigated the impact of tumor on severity of vascular inflammation following IR. Hypofractionated partial brain gamma radiation was used with dose of 40 Gy (8 Gy/fraction/day, 5 days a week) on healthy or tumor-bearing rats. A significant increase in the number of adherent leukocytes in irradiated tissue was found in tumor-bearing mice only,^44^ showing that the presence of a tumor could worsen radiation-induced vascular inflammation.

### Timing of Effects

We were interested to determine the timing of vascular and neurocompartment effects following IR to deduce if vascular effects precede tissue effects or vice versa. Figure 4E shows the majority of studies (84.1%) investigated changes in the acute time window (<1 month), relative to 41.5% and 36.6% in the delayed (1–3 months for preclinical or 1–6 months for clinical) and late (≥3 months for preclinical or ≥6 months for clinical) windows, respectively. As such, there is some inherent selection bias in the reporting of the results favoring acute effects. Table 1 summarizes IR effects from all studies categorized into acute, delayed, and late time windows for preclinical and clinical studies. A more detailed version of Table 1, including individual study characteristics arranged into NVU components is given in Supplementary Tables 1 and 2.

**Table 1.** Summary of NVU Changes at Acute, Delayed and Late Timepoints

(A) Preclinical studies			
	Acute effects: during to < a month post-IR	Delayed effects: from 1 to 3 months post-IR	Late effects: ≥3 months post-IR
Endothelial layer			
*Vascular permeability and transport*	Increased permeability to horseradish peroxidase (HRP, 144 kD),^36^ Evans blue (EB, 0.96 kDa),^20^ Immunoglobulin G (IgG, ~150 kDa) and albumin (~67 kDa),^37^ sulforhodamine B (SRT, 0.58 kDa),^32^ a 4.4 kDa fluorescein isothiocyanate (FITC) dextran,^41^ a 0.56 kDa Gd-DOTA,^43^ a 3 kDa Texas-Red-dextran,^44^ a Gd-chelate,^48^ Gd-DTPA.^49^ No extravasation of a 70 kDa (FITC-dextran),^32^ a 4 kDa FITC-dextran and a 70 kDa Rhodamine B-dextran,^21^ a 3.5 kDa gadolinium-based P846 molecule,^43,51^ IgG,^52^ and EB,^45,47^ Gd-DOTA.^22^ A 60% reduction in P-glycoprotein density,^63^ a 9% drop in apparent diffusion coefficient (ADC),^121^ increased water content in irradiated brains,^54,121^ increased ADC values, a transient reduction in glucose (FDG) uptake and expression of GLUT 1 and GLUT 3.^122^ No significant changes in water content,^47^ cerebral blood flow,^123^ and no T2W hyperintensities.^49^	Increased vascular permeability to EB,^20,45,47^ IgG,^40^ Gd-DTPA, and fibrin.^49^ No leakage of EB,^20^ a 70 kDa FITC-dextran,^32^ a 4.4 and 38.2 kDa FITC molecules,^39^ a 4 kDa FITC-dextran and a 70 kDa RhodamineB-dextran,^21^ a 3.5 kDa gadolinium-based P846 molecule,^51^ and IgG.^52^ Increased water content in irradiated brain,^47^ increased ADC values and glucose uptake.^123^ Reduced uptake of glucose (FDG) and11C-MeDAS.^124^ A hyperintense T2 signal followed by hypointensities.^49^	Increased extravasation of HRP,^30,36^ EB,^20,47^ Gd-DTPA,^38^ FITC-dextran molecules (4.4 and 38.2 kDa),^39^ IgG,^40^ albumin,^42^ Gd-DOTA,^22^ Gd-DTPA, and blood fibrin.^49^ No extravasation of IgG,^52^ HRP and EB.^50^ No significant changes on water content,^47^ and uptake of11C-MeDAS.^124^ Reduced uptake of glucose (FDG).^124^ A hyperintense T2 signal followed by hypointensities.^49^
*EC density and viability*	Decreased EC division,^20^ EC loss,^47,62^ increased intracellular ROS production,^57^ significant loss of viable ECs.^56^ No effect on ECs.^51^	Decreased EC division,^20^ EC loss,^51,62^ significantly increased number of CD31+ cells.^47^	Decreased EC division,^20^ reduced EC number,^62,67^ EC density similar to controls affected.^47^
*Ultrastructure*	Nuclear and cytoplasmic swelling,^68^ an intact endothelium,^36^ no effect on EC structure.^47,81^	No effect on EC structure.^47^	An intact endothelium,^36^ shorter and less dense EC tight junctions, and increased EC vesicular activities,^39^ abnormal ECs,^47^ nuclear and cytoplasmic swelling.^50^
*Capillary morphometry*	A slight extravascular space enlargement,^81^ vasogenic edema,^65,66^ perivascular lymphocytes and plasma cells,^41,44,66^ reduced blood flow and volume,^71^ fibrin deposition and vessel wall thickening,^20^ increased vessel size index,^51^ increased vessel volume.^61^ No significant changes on vessel^32,36,49,60^, blood flow^123^, and blood volume.^51^	Vasogenic edema,^65^ vessel dilation,^42^ reduced cerebral blood flow,^71^ a disorganized vascular network,^51^ increased blood vessel volume.^61^ No significant changes on vessel^32,39,49,60^ or blood flow.^123^	Thickened vessel wall,^60,70^ vasogenic edema,^65^ abnormal angiogenesis,^30,49^ fibrin deposition in capillary wall,^20^ vessel dilation and tortuosity,^49,56^ and perivascular edema.^22,36,47,49^
*Surface proteins*	Upregulated expression of ICAM-1,^42,52–56^ ICAM-1/COX,^54^ ICAM-1/PAI-1,^57^ ICAM-1/TNF expression,^41^ thrombomodulin,^59^ and VCAM-1.^56^ Reduced expression of E-selectin,^59^ and vWF expression,^60^ increased leukocyte adhesion to endothelium,^41,44^ and upregulated eNOS immunoreactivity.^61^	Increased ICAM-1 expression.^42^Reduced vWF expression,^60^ and an increased eNOS immunoreactivity.^61^	Increased expression of ICAM-1,^42^ CD31.^45,47^ Reduced expression of vWF.^60^
*Junctional proteins*	Reduced expression of ZO-1,^60^ claudin 5, and occludin.^61^	Reduced expression of ZO-1,^60^ occludin/lectin, and claudin 5/lectin.^61^	Shortened and less dense TJs,^39^ and decreased ZO-1 expression^60^
*Gene expression and Signaling pathways*	Upregulated genes that are key in increasing cell death and BBB permeability, and downregulated genes vital in processes like cell cycle regulation, learning, and memory.^42^	Not studied.	Not studied.
Extracellular matrix			
*Structure and proteins/lipids/sugars*	Significant upregulation of MMP-2, MMP-9 and TIMP-1 expression levels but not TIMP-2,^78^ loss of collagen IV,^78^ and proteoglycans,^76,77^ a slight increase in CD147 and MMP-9 expression.^45^ Collagen IV not affected.^21^	Increased expression of CD147 and MMP-9.^45^ Collagen IV not affected.^21^	Thickened basement membrane,^47,50^ cytoskeletal component loss,^36^ increased expression of CD147, and MMP-9.^45^
Pericytes			
*Cell density and structure*	A 17% decrease in number of pericytes,^69^ no effect on pericytes.^81^	Nonsignificant loss of pericytes.^69^	Not studied.
Astrocytes and microglia			
*Cell number and viability*	Loss of astrocytes^32,37,89,90^ and microglia,^92,96^ reduced glia cell proliferation and viability.^77^	Increased number of dividing astrocytes,^20^ no significant microglial loss.^92^	Not studied.
*Cell structure*	No effect.^47,81^	No effect.^47^	Astrocyte hypertrophy.^20,47,50^
*Surface protein expression*	Increased expression of GFAP^20,37,41,44,52–54,65,66,94^ and IBA-1,^52,96,113^ significantly increased amount and activity of intracellular tissue plasminogen activators, and extracellular type IV collagenase levels in astrocytes.^88^ Upregulated expression of PTK-3,^125^ and GFAP/VEGF,^60^ reduced bFGF mRNA levels in astrocytes.^126^ No significant increases in TNF-a production by both astrocytes and microglia cells,^117^ no gliosis.^21^	Increased expression of GFAP,^20,39,49,52,65,66,95^ IBA-1,^49,66,113^ GFAP/VEGF,^60^ IBA1+/C1q, GFAP+/C3, and TLR4/GFAP.^95^ No gliosis.^21^	Increased reactivity of astrocytes^20,22,39,45,49,50,52,66,93^ and microglia.^22,49^ Increased GFAP/VEGF expression.^60^ No gliosis.^21^
Neurons and oligodendrocytes			
*Neuronal cell density and viability*	Lost neurons,^32,89,90,102–105^ increased number of undifferentiated neurons,^94^ reduced neuronal proliferation,^106,113^ increased oxidative stress, DNA damage, and reduced metabolic activity. Neuronal growth not affected.^21^	Neuronal loss,^20,102,103,105,107^ reduced neuronal proliferation.^106^ Neuronal growth not affected.^21^	Neuronal loss,^20,101,103^ reduced neuronal proliferation.^127^ Neuronal growth not affected.^21^
*Protein expression and cell signaling*	A significant reduction in levels of BDNF and TrkB neurotrophic factors, and H3 acetylation.^106^ Activated intracellular signaling pathways like Akt and MAPK, and an increased expression of neurotrophins.^114^	Increased enolase expression,^65^ a significant reduction in levels of BDNF and TrkB neurotrophic factors.^106^	Not studied.
*Neural tissue structure*	Myelin synthesis inhibition,^110^ demyelination,^65^ tissue necrosis,^31,37,65,102,105,109^ myelin sheath swelling,^66^ infiltration of leukocytes,^31^ macrophages,^37^ and neutrophils.^52^ Increased tortuosity, nonspecific uptake, and extracellular space volume fraction,^37^ condensed neuronal nuclei.^37^ No necrosis.^52^	Necrosis,^20,40,49,65^ demyelination,^20,65,102,105,107,112^ myelin sheath swelling,^66^ infiltration of lymphocytes and dendritic cells.^52,66^ No necrosis.^52^	Necrosis,^20,22,40,49,60,62,65,93,103^ demyelination,^20,22,42,65,93,103,111^ myelin sheath swelling,^36,42^ synaptic damage and neuropil mitochondrial alterations,^36^ infiltration of lymphocytes and dendritic cells,^20,52^ neuronal chromatin condensation,^47^ cavitation and mineralization.^22^ No demyelination,^118^ no necrosis.^52,118^
*Oligodendrocyte cell number and structure*	Lost oligodendrocytes.^103,109^ No effect on oligodendrocytes.^81^	Lost oligodendrocytes.^103^	Lost oligodendrocytes.^103^ Oligodendrocytes not affected.^118^
Changes nonspecific to any NVU component	Reduced mitotic cells,^70,90,103^ nuclei piknosis,^70,113^ cell number reduction,^90,128^ apoptosis,^32,61,96,102,111,114,129,130^ upregulated VEGF expression,^44,51,72^ increased DNA damage,^113^ no significant apoptosis,^44^ increased CXCL12 expression.^72^ Significant increase in expression of Ang-2, but significantly decreased Ang-1 and Tie-2 expression levels.^60^	Nuclei piknosis,^70^ cell number reduction,^128^ reduced proliferating cells.^103^ Increased VEGF expression.^47^ Significant increase in expression of Ang-2, but significantly decreased Ang-1 and Tie-2 expression levels.^60^	Nuclei piknosis,^70^ cell reduction,^128^ reduced cell proliferation.^101,103^ Increased VEGF expression.^47^ Significant increase in expression of Ang-2, but significantly decreased Ang-1 and Tie-2 expression levels.^60^

(B) Clinical studies			
	Acute effects: during to < a month post-IR	Delayed effects: from 1 to 6 months post-IR	Late effects: ≥6 months post-IR
Endothelium			
*Vascular permeability*	Significantly increased permeability to 20 mCi Technetium-99m-glucoheptonate tracer,^29^ to a 10 kDa dextran tracer.^46^	Not studied.	Slight permeability to 20 mCi Technetium-99m-glucoheptonate tracer.^29^
*EC number and viability*	Increased lactate dehydrogenase activity, but no increase in cell death.^58^	Not studied.	Not studied.
*Surface proteins*	Upregulated expression of ICAM-1 and E-selectin.^58^ A transient reduction in PECAM-1 levels.^46^	Not studied.	Not studied.
*Junctional proteins*	A significant decrease in trans-endothelial electrical resistance values.^46^	Not studied.	Not studied.
Astrocytes			
*Cell viability*	Short-term increase in DNA damage.^91^	Not studied.	Not studied.
Neurons			
*Cell density and viability*	Increased neuronal death, oxidative stress, DNA damage and undifferentiated neurons, reduced neuronal proliferation, and metabolic activity.^108^	Not studied.	Not studied.

#### Timing of vascular effects.—

Vascular effects were observed in acute, delayed, and late time intervals. Most vascular effects observed in delayed and late windows were also observed in acute windows, a suggestive of early damage that is chronic or permanent. The following summarizes key changes that were observed at all 3 time intervals: increased BBB permeability to small and large molecules; focal hyperintensities on T2-MRI; upregulated expression of ICAM-1 and downregulated expression of vWF; reduction in junctional proteins, particularly ZO-1; thickened vessel wall, perivascular edema, and angiogenesis; reduced uptake of glucose into brain tissue; increased glial reactivity. Gene expression changes in ECs were observed at acute timepoints only, although they were not studied at delayed or late timepoints. EC loss was observed at acute and delayed timepoints. EC loss data at late timepoints were mixed. Nuclear and cytoplasmic swelling of ECs was observed at acute and late timepoints and not studied at delayed timepoints. P-glycoprotein expression and GLUTs were reduced at acute timepoints only. Astrocyte and pericyte loss were predominantly observed at acute and delayed timepoints; however, there were a lack of studies investigating their damage at late timepoints.

#### Timing of neurocompartment effects.—

Similar to vascular effects, most neurocompartment effects observed in delayed and late time intervals were also present at acute timepoints. The following were observed at all timepoints: increased ECM expression of CD147 and MMP-9; neuronal loss; myelin loss; necrosis; peripheral immune cell infiltration; and reduced number of oligodendrocytes.

## Discussion

The purpose of this systematic review was to summarize published data on radiation-induced effects on the BBB and wider NVU of normal brain tissue following IR. There were 2 key aims: to understand the factors that influence the magnitude of changes and to determine the timing of vascular changes in relation to neurocompartment changes.

Overall, IR was found to cause widespread and varied impacts on NVU components. Unfortunately, only a small number of studies could be included when investigating the factors driving NVU damage severity; most studies were not comparable due to the high variability in IR characteristics, study endpoints, and follow-up timelines. Despite this, we were able to draw some meaningful conclusions. The impact of dose was the most widely investigated factor. Almost all studies concluded that increasing dose causes more severe damage, as expected, which often manifested as an earlier presentation of pathology. This was true for both vascular and neurocompartment NVU components. The other factors including high/low LET, fractionation, dose rate, beam size, and presence of a tumor were less studied. A single study directly compared high LET and low LET radiation types, and found more impact with low-LET. Two studies directly compared the impact of dose rate and found that lower dose rates led to greater effects on junctional proteins, eNOS, IBA1+/C1q co-labeling, TLR4 expression, and TLR4+/GFAP+ cells. Only one study investigated fractionation versus single dose on MMPs and TIMPs, and while changes were observed for both methods, the authors could not find substantial differences between them. One study with similar doses investigated broad versus focused (micro) beams and found less impact of microbeams on BBB leakage and demyelination. Finally, one study investigated IR effects in mice with and without a tumor. They found that tumor-bearing mice exhibited a greater degree of leukocyte adherence, indicating increased inflammatory response of vessels to IR. These studies hint at factors that influence the severity of normal tissue effects, but there is a clear need for more prospective studies that compare IR effects with dose rate, fractionation schemes, beam width, low/high LET, and naive/tumor-bearing models.

While not assessed as a key factor during analysis, the volume of the brain irradiated and that subsequently affected by IR was noted. Figure 4B summarizes the number of studies using WBI (*n* = 34), PBI (*n* = 34), and PBI + WBI (*n* = 1) (also see Supplementary Tables 1 and 2). Compared to PBI, WBI generally induced more severe effects on NVU structures.^107^ Of the 35 PBI studies, 11 reported changes in the irradiated and nonirradiated hemispheres,^30,37,38,40,44,53,63,65,81,102,123^ indicating the non-local effects of IR. Lower IR doses,^40^ high LET radiation types (such as BNCT,^30^ PRT,^49^ and neutrons^81,122^), gamma rays^20,31,45,47,50,71,93,113^ (as compared to x-rays), and more focused radiation beams (such as MRT^32,43,48,51,89,121^ and MSB^21^) showed no or mild-delayed impacts in opposite hemispheres.

**Figure 6.**
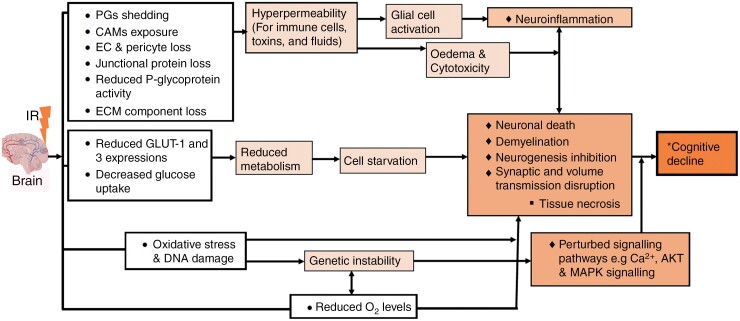
Radiation-induced NVU changes and possible biological processes that promote cognitive decline. Observations from the above studies provide clues on their possible linkage to some of the biological processes reported in neurodegenerative studies.

Data synthesized during this systematic review highlights that both vascular and neurocompartment changes can occur during acute, delayed, and late time periods, often beginning in the acute time interval. The apparent chronic nature of these changes indicates that brain tissue following irradiation is likely to be in a chronic neuroinflammatory state with a sustained inadequate supply of nutrients. It is likely, therefore, that vascular changes could contribute to cognitive dysfunction, which typically occurs as a late effect. While we did not include “cognition” or similar in our search terms, 5 studies were identified that assessed cognitive function (all in non-tumor-bearing rodents) alongside radiation-induced NVU changes. They reported reduced working and spatial memory at 3 months post-IR,^101^ at 6 months post-IR,^42^ at 1-month post-IR,^106^ and from 2 to 3 months post-IR.^124^ One study observed reduced locomotor activities from day 1 to 5 months post-IR,^109^ and all these cognitive deficits were associated with NVU changes, such as myelin loss, increased BBB permeability, and reduced number of neurons. Figure 6 synthesizes observed NVU changes, and how vascular changes observed in this review may contribute to cognitive decline.

## Limitations and Current Gaps in Knowledge

Most studies were done in rodents (89%) using partial or whole brain irradiation, mostly at large single doses (Figure 4). This clearly differs from the therapeutic management of brain tumors in the clinic, and does not capture the differences in management of different patients (pediatric, adult, and tumor location). The small number of clinical studies identified means it is unclear how these results translate into clinical settings, where lower fractionated doses are used. There were no studies investigating sex-dependent IR effects on NVU components; however, 4 groups examined the age-dependent effects, all in rodents^92,96,102,109^ (see Supplementary Material for details on these studies).

The predominant model used was non-tumor-bearing rodents; out of the 69 in vivo studies, 61 used healthy (non-tumor-bearing) subjects. This means that it is currently uncertain how the tumor (and/or tumor resection) impacts normal tissue toxicity, and vice versa. It is also not clear how the tumor’s presence (or resected volume) will interact with cognition, in particular the long-term changes. In the clinic, it is difficult to quantify/characterize cognitive effects stemming from RT treatment since >90% of brain tumor patients suffer from cognitive deficits pretreatment^131–134^ due to the volume and invasion effects of the tumor itself, including hydrocephalus before diagnosis.^135^ However, cognitive impairment has been reported in subjects exposed to IR both in the presence^85,136–141^ or absence of tumors,^42,101,106,109,124,142^ suggesting that normal tissue changes resulting from IR exposure can contribute to cognitive dysfunction independent of the tumor. To fully address whether normal tissue toxicities drive long-term neurocognitive changes, independently of the tumor, studies are needed that compare IR effects and behavioral outcomes in tumor-bearing and naive models. Incorporating in vivo imaging techniques, such as MRI, that can show NVU changes over time in the same animal/subject^143^ may provide a better understanding of when normal tissue changes in relation to changes in cognition, helping to identify treatment targets.

Radiation effects were primarily reported in the cortex^20–22,29,30,32,36,38,39,41,44,47,49,51,52,63,66,76–78,89,103,105,107,109,121–124^ and the hippocampus,^22,40,42,61,69,76,78,92,95,96,105,106,113,114,122–124,144^ however, this could partially reflect selection bias, since studies tended to search for changes in locations known to play key roles in cognitive function. Apart from being located in the path of almost all radiation beams, the cerebral cortex accounts for 82% of the brain mass,^145^ thus, is likely to receive a substantial dose for most RT plans. However, the cortex performs many different functions depending on the location, so specific structures irradiated will likely affect different cognitive functions/domains. For the hippocampus, its selection in studies might be due to its role in the formation of major brain tissue cells, the neurons. In adulthood, neurogenesis occurs only in 2 niches: the SVZ of the lateral ventricles and in the SGZ in the DG of the hippocampus.^146,147^ Thus, investigating changes in those zones is key since neurogenesis positively regulates cognitive function. A wider evaluation of radiation-induced damage across the entire brain is needed, but this comes with statistical challenges associated with multiple comparisons.^148^

Another important limitation of this review is that we focused on RT, yet this treatment is often given in synergy with other treatments, such as surgery, chemotherapy, and immunotherapy in clinical settings. Even though our results reveal non-confounded effects of IR on the normal brain tissue, they show an incomplete picture.

## Conclusions

We have summarized evidence showing radiation-induced structural, functional, cellular, protein, and gene expression changes in vascular and neural components. The severity of changes was worst for low LET radiation, higher doses, conventional dose rates, and when a tumor was present in the irradiated region. However, data directly comparing these effects were sparse, and more should be done to systematically evaluate the impact of these factors on normal tissue damage. Conducting more research with FLASH IR, high LET radiation, and focused beams should be given attention due to their potential in sparing the surrounding normal tissues.

Our second aim was to establish when vascular changes occur in relation to neurocompartment changes. We found radiation led to widespread and prolonged vascular changes (acute, delayed, and late) that matched the timing and duration of neurocompartment changes. A small number of studies that assessed cognitive impairment found good relationships with NVU changes. These findings indicate that vascular changes could produce long-lasting vascular and NVU dysfunction that promotes neurocognitive decline via processes such as chronic neuro-inflammation and neurovascular uncoupling.

## Supplementary Material

vdae098_suppl_Supplementary_Materials

vdae098_suppl_Supplementary_Table_S1

vdae098_suppl_Supplementary_Table_S2
