# Supplementary material for: A systematic review of normal tissue neurovascular unit damage following brain irradiation—Factors affecting damage severity and timing of effects
Source: Neurooncol Adv. 2024 Jun 13;6(1):vdae098. doi: 10.1093/noajnl/vdae098 (PMC11375288; doi:10.1093/noajnl/vdae098)
Supplement: vdae098_suppl_Supplementary_Materials [file vdae098_suppl_supplementary_materials.docx]

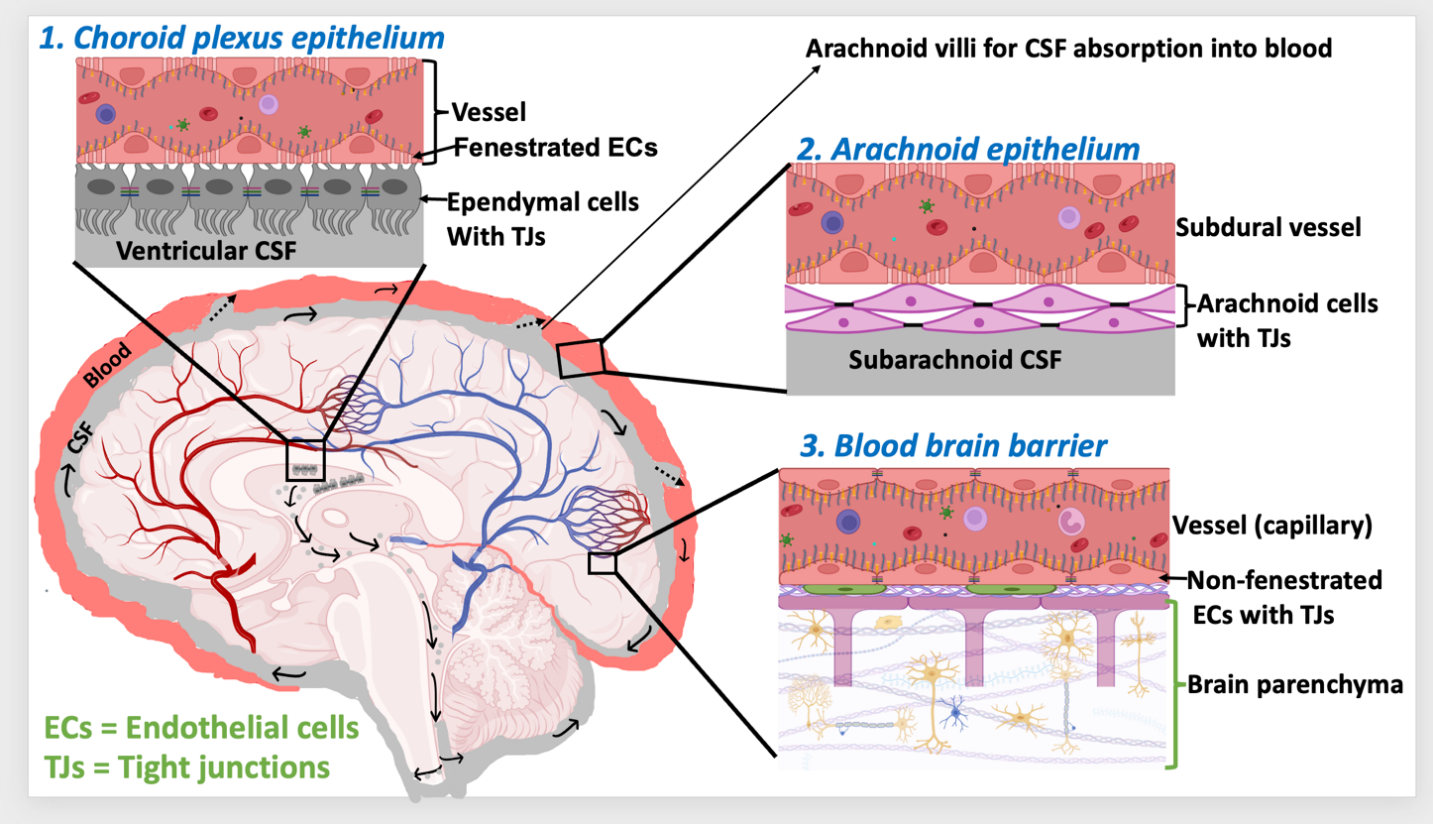


**Supplementary figure 1. The three main brain barriers that maintain the central nervous system (CNS) homeostasis.** *The choroid plexus epithelium* (in the choroid plexus) is made by tight-junctioned ependymal cells separating blood from the ventricular cerebrospinal fluid (CSF). The *arachnoid epithelium* (in the meninges) is made by tight-junctioned arachnoid cells separating mainly venous blood from subarachnoid CSF^1^. The blood brain barrier (BBB) (within the brain tissue) has non-fenestrated, tight-junctioned endothelial cells (ECs) separating blood from the brain parenchyma, and is the major and an evolutionarily conserved component of the CNS blood vessels^1^ ^2^. It is the capillary structure that allows selective exchange of molecules and substances between blood and the brain parenchyma to protect the brain tissue from circulating pathogens and toxins, thus ensuring a proper neuronal function and a stable CNS homeostasis^3^ ^4^. The integrity of the BBB is maintained by the collective effort of all the neurovascular unit (NVU) components (Figure 2), but as we age, the BBB naturally becomes leaky regardless of one’s health condition^5^ ^6^. (Figure created with BioRender.)

*Age effects*

Fukuda et al. exposed partial brains of nine day-(P9)-and-23-day-(P23)-old rats to a single 8 Gy dose of photons and examined NVU changes at 6 h, 7 days and 10 weeks after IR^148^. By 6 h post-IR, they observed an increased activation of caspase-3, p53, and nitrotyrosine in SVZ and DG of both hemispheres in P9 compared to P23 and control groups. Also, numbers of nestin+ and doublecortin+ cells in SVZ and DG of both hemispheres significantly dropped by ≥ 37 % (in P9) and ≥ 28 % (in P23) at all time points compared to controls. When Blomstrand et al. irradiated 9-day-old (P9) and 6-months-old rats with a single WBI dose (6 Gy) of photons, they observed a short-term (from 6 h to 1-week post-IR) significant increase in hippocampal caspase-3 activity (by 467%) in P9 rats, but it was not significantly changed in 6-months-old or control rats at all time points (6 h, 7 days and 4 weeks)^137^. However, by 4 weeks post-IR, microglia density in juvenile DG increased by 12 % and decreased by 35 % in the adult DG. A similar WBI study that exposed 8-and-28-months-old rats to a single gamma-ray dose of 10 Gy observed a 20 % to 25 % decrease in hippocampal microglial density in both ages within 1-week post-IR, but by 10 weeks, levels were similar to those of controls^142^. There was one study that examined neural structure effects between 1 day and 5 months after irradiating whole brains of differently aged mice (3, 4, and 8-weeks-old) with a single gamma-ray dose of 25 Gy^154^. They observed a transient (up to 2 weeks post-IR) significant loss of oligodendrocytes and myelin in cortex and corpus callosum (of treated 3-weeks-old group), and in white and gray matter (of treated 4-weeks-old group) compared to controls. In the 8-weeks-old group, demyelination was worse than in younger groups at 2 weeks post-IR, and no remyelination was seen up to four months later. These data show that younger rats are more sensitive brain irradiation compared to older rats. On the other hand, neural tissue changes appear to be more severe in older mice compared to young ones. Therefore, more studies will be needed to further clarify how IR affects differently aged rodent species.

References

**1.** Abbott NJ. Evidence for bulk flow of brain interstitial fluid: significance for physiology and pathology. *Neurochem Int.* 2004; 45(4):545-552.

**2.** Kadry H, Noorani B, Cucullo L. A blood-brain barrier overview on structure, function, impairment, and biomarkers of integrity. *Fluids Barriers CNS.* 2020; 17(1):69.

**3.** Sanchez-Cano F, Hernández-Kelly LC, Ortega A. The Blood-Brain Barrier: Much More Than a Selective Access to the Brain. *Neurotox Res.* 2021; 39(6):2154-2174.

**4.** Begley DJ, Brightman MW. Structural and functional aspects of the blood-brain barrier. *Prog Drug Res.* 2003; 61:39-78.

**5.** Montagne A, Barnes SR, Sweeney MD, et al. Blood-brain barrier breakdown in the aging human hippocampus. *Neuron.* 2015; 85(2):296-302.

**6.** Verheggen ICM, de Jong JJA, van Boxtel MPJ, et al. Increase in blood-brain barrier leakage in healthy, older adults. *Geroscience.* 2020; 42(4):1183-1193.
