# Supplementary material for: A systematic review of normal tissue neurovascular unit damage following brain irradiation—Factors affecting damage severity and timing of effects
Source: Neurooncol Adv. 2024 Jun 13;6(1):vdae098. doi: 10.1093/noajnl/vdae098 (PMC11375288; doi:10.1093/noajnl/vdae098)
Supplement: vdae098_suppl_Supplementary_Table_S1 [file vdae098_suppl_supplementary_table_s1.docx]

**Supplementary table 1 | Evidence of radiation-induced neurovascular unit changes/damage in rodents, canines, and rabbits**

| **Author, year** | **Subject model, Sex, Age** | **IR type/ modality**  **& dose** | **Fraction,**  **brain volume** | **Brain region** | **Assay used** | **Follow-up time after IR** | **Acute effects: during to < a month post-IR** | **Delayed effects: from 1 to 3 months post-IR** | **Late effects: ≥ 3 months post-IR** |
| --- | --- | --- | --- | --- | --- | --- | --- | --- | --- |
| **Endothelial layer** | | | | | | | | | |
| *Vascular permeability and transport* | | | | | | | | | |
| Coderre et al., 1994 | Rat implanted with 9L gliosarcomato left side, n = 7, Sex and age: N/A | Boron neutron capture therapy (BNCT); 0 or 13.4 Gy  OR  X-rays;  22.5 Gy to the tumour | Single,  Partial brain irradiation (PBI) | Cortex, Corpus callosum (CC), Hippocampus, Striatum | Histochemistry | 1 year | Not studied. | Not studied. | Slight horse radish peroxidase (HRP, 144 kD) leakage in ipsilateral hemisphere after BNCT, but intense HRP leakage in both hemispheres after x-rays. |
| Cicciarello et al., 1996 | Rat, n = 8, M, 280-320 g | Photons;  0 or 40 Gy (2 Gy/fraction/day, 5 days a week) | Fractionated,  Whole brain irradiation (WBI) | Cortex | Histochemistry | 15 and 90 days | Increased HRP extravasation in irradiated cortices compared to non-irradiated control brains. | Not studied. | Increased HRP extravasation in irradiated cortices compared to non-irradiated control brains. |
| Kamiryo et al., 1996 | Rat, n = 21, M, 250-300g | Gamma knife surgery (GKS); 0, 100, 120, 140, 160, 180, or 200 Gy | Single,  PBI | Cortex | Histochemistry | 1 month | Not studied.  . | Significant Evans blue (EB, 0.96 kDa) leakage in irradiated side post all doses. | Not studied. |
|  | Rat, n = 21, M, 250-300g | GKS; 0, 50, 75 or 120 Gy | Single,  PBI | Cortex | Histochemistry | 1 to 4 weeks (120 Gy),  1 to 6 months (75 Gy), and 1 to 12 months (50 Gy) | Increased EB leakage in irradiated region after 120 Gy. | No EB leakage after 50 or 75 Gy. | Increased EB leakage in in irradiated region after 75 Gy but not 50 Gy. |
| Sykova et al., 1996 | Rat pups, n = 63, Sex: N/A, 1-day-old | X-rays; 0, 20 or 40 Gy | Single,  PBI | Somatosensory cortex, White matter | Histochemistry | 20 days | Immunoglobulin G (IgG, ~150 k Da) and albumin (~67 kDa) extravasation in both sides, but predominant in ipsilateral side of 40 Gy. | Not studied. | Not studied. |
| Karger et al., 1997 | Rat, n = 66, M, 2.25-months-old | Gamma-rays; 0, 20, 30, 40, 50, or 100 Gy | Single PBI | Whole brain | Dynamic contrast enhanced- magnetic resonance imaging (DCE-MRI) with Gd-DTPA tracer. | 19 months | Not studied. | Not studied. | Gd-DTPA tracer leakage from 5 to 13 months post-100 Gy, and at 19 months post-doses <100 Gy. |
| Kamiryo et al., 2001 | Rat, n = 6, M, 250 to 300 g | GKS; 0 or 75 Gy | Single PBI | Cortex | EM | 3.5 months | Not studied. | Not studied. | No extravasation of HRP and EB in ipsilateral side compared to non-irradiated side. |
| Serduc et al., 2006 | Mouse, n = 55,  Sex: N/A,  5-weeks-old | X-ray microbeam radiation therapy (MRT); 0, 312 or 1000 Gy. | Single PBI | Cortex, Hippocampus | Histochemistry, Two-photon microscopy | 3 months | Sulforhodamine B (SRB, 0.58 kDa) extravasation in ipsilateral side up to 12 days post-1000 Gy. No leakage of Fluorescein iso-thiocyanate (FITC-dextran, 70 kDa) in both doses. | No FITC-dextran extravasation in both doses. | Not studied. |
| Yuan et al., 2006 | Mouse, n = 46, M, 6-to-7-weeks-old | X-rays; 0 or 40 Gy (2 Gy/day, 5 days/week) | Fractionated WBI | Cortex,  Hippocampus | Two-photon microscopy, Histochemistry | 180 days | Not studied. | No leakage of 4.4 and 38.2 kDa FITC molecules. | Increased extravasation of FITC- dextran molecules (4.4 and 38.2 kDa) from day 90 to end (p < 0.05). |
| Ernst-Stecken et al., 2007 | Rat, n = 9, M, Adult | X-rays; 0, 20, 30 or 40 Gy (10 Gy/ fraction/week) | Fractionated  PBI | Hippocampus, Subventricular zone (SVZ) | MRI (4.7T), Computed tomography (CT), Histochemistry | 16 weeks | Not studied. | Mild IgG extravasation in ipsilateral side of 30 Gy. Significant IgG leakage in SVZ in both brain sides after of 40 Gy. | Moderate IgG extravasation in irradiated side of 30 Gy. Significant IgG leakage in SVZ in both brain sides after of 40 Gy. |
| Ricard et al., 2009 | Mouse, n = 24, F, Age: N/A | Monochromatic synchrotron X-ray beams (MSB); 0 or 15 Gy | Single PBI | Cortex | Two-photon microscopy | 1 month | No extravasation of a 4 kDa FITC-dextran and a 70 kDa RhodamineB-dextran dyes. | No leakage of a 4 kDa FITC-dextran and a 70 kDa RhodamineB-dextran dyes. | Not studied. |
| Wilson et al., 2009 | Mouse, n = 5, M, 8-to-10-weeks-old | X-rays; 0 or 20 Gy | Single  PBI | Cortex | Photon microscopy | 2 days | Increased FITC-dextran (4.4 kDa) extravasation. | Not studied. | Not studied. |
| Bouchet et al., 2010 | Rat implanted with 9L gliosarcoma, n = 59, Sex: N/A, 10-weeks-old | MRT; 0, 350 (unidirectional) or 700 Gy (bidirectional/crossfired) | Single  PBI | Caudate nucleus | MRI with a Gd-based 3.5 kDa P846 tracer | 45 days | No P846 extravasation in both doses. | No P846 extravasation in both doses. | Not studied. |
| Lemasson et al., 2010 | Rat implanted with 9L gliosarcoma, n = 20, M, 120-150 g | MRT; 0 or 400 Gy, given in two arrays | Single  PBI | Caudate nucleus | DCE-MRI (4.7 T) with 3.5 kDa P846 and 0.56 kDa Gd-DOTA tracers | 8 days | Only Gd-DOTA significantly leaked in the ipsilateral normal tissues. | Not studied. | Not studied. |
| Wu et al., 2010 | Mouse, n = 48, M, 10-weeks-old | X-rays; 0, 10, 12 or 17 Gy | Single  WBI | Whole brain | Histochemistry | 8 months | Not studied. | Not studied. | An increased albumin extravasation in 10 Gy. |
| Moravan et al., 2011 | Mouse, n = 45, M, 8-to-10-weeks-old | Gamma-rays; 0 or 35 Gy | Single  WBI | Striatum,  White matter | Histochemistry | 1 year | No IgG extravasation. | No IgG extravasation. | No IgG extravasation. |
| Zawaski et al., 2012 | Rats bearing tumours, n = N/A, M, 6-to-8-weeks-old | Gamma-rays; 0 or 40 Gy (8 Gy/fraction/day) | Fractionated  PBI | Cortex | Photon microscopy | 11 days | Increased permeability to Texas-Red-dextran (3 kDa) in ipsi-and-contralateral normal tissues, but not in non-irradiated controls. | Not studied. | Not studied. |
| Li et al., 2013 | Rat, n = 76, M, 200-240 g, | Gamma-rays; 0 or 75 Gy | Single  PBI | Cortex | Histochemistry | 12 weeks | No EB dye extravasation. | Increased EB staining (by 2-fold) in ipsilateral side only (p < 0.001). | Not studied. |
| Cheng et al., 2014 | Rat, n = 96, M, 200-240 g | Gamma-rays; 0 or 60 Gy | Single  PBI | Cortex | Histochemistry | 24 weeks | No EB leakage seen. | Increased EB extravasation in irradiated cortex. | Increased EB extravasation in irradiated side up to 24 weeks post-IR. |
| Bouchet et al., 2017 | Rat hemi-implanted with F98 tumour cells, n = 18, sex; N/A, 10-weeks-old | X-ray broad beams (BB);  0 or 10.5 Gy  OR  MRT; 241.4-Gy | Single  PBI | Whole brain | MRI (4.7 T) with Gd-chelate tracer | 14 days | MRT induced a significant ipsilateral increase in Gd-chelate extravasation (up to 1 week) compared to BB-irradiated tissues (p = 0.0085) and controls (p = 0.0242). | Not studied. | Not studied. |
| Prezado et al., 2017 | Rat, n = 23, sex; N/A,  1.5 months | Conventional/BB protons (PRT)  Or proton minibeam RT (pMBRT); 0 or 25 Gy | Single  WBI | Whole brain | MRI (7T) with Gd-DOTA,  Histochemistry | 6 months | No Gd-DOTA extravasation after IR. | Not studied. | Only PRT induced Gd-DOTA extravasation. |
| Suckert et al., 2021 | Mouse, n = 80, F, 11-to-13-weeks-old | Protons; 0, 45, 65 or 85 Gy (C57BL/6 Mouse)  OR  0, 40, 60 or 80 Gy, (to C3H/He mice) | Single  PBI | Hippocampus | MRI (1T), Histochemistry | 160 days | A dose dependant leakage of Gd-DTPA in ipsilateral sides only, especially in C3H/He mice. | A dose dependant leakage of Gd-DTPA in ipsilateral sides only. | A dose dependant leakage of Gd-DTPA and fibrin in ipsilateral sides only. |
| Mima et al., 1999 | Rat, n = 12, M, 4-to-5-weeks-old | X-rays; 0 or 25 Gy | Single  PBI | Cortex | Western blotting, Histochemistry | 5 days | A 60% and 15% reduction in P-glycoprotein density in ipsi-and-contra-lateral sides, respectively, compared to controls. | Not studied. | Not studied. |
| Moore et al., 2004 | Mouse, n = 78, sex; N/A, 6-to-8-weeks-old | Gamma-rays; 0 or 35 Gy | Single  WBI | Cortex | Dry/wet weight method | 1 day | An increased water content in irradiated brains compared to controls (p < 0.05). | Not studied. | Not studied. |
| Serduc et al., 2008 | Mouse, n = 50, F, 5-weeks-old | MRT; 0, 312 or 1000 Gy | Single  PBI | Cortex | Diffusion-weighted (DW) MRI (7T) | 4 weeks | A 9% drop in apparent diffusion coefficient (ADC) in ipsilateral cortex (up to 1 day) of 312 Gy only. A transient 0.7 % rise in tissue water content in the ipsilateral side of 1000 Gy. | Not studied. | Not studied. |
| Cheng et al., 2014 | Rat, n = 96, M, 200-240 g | Gamma-rays; 0 or 60 Gy | Single  PBI | Cortex | Dry/wet weight method | 24 weeks | No effect on water content | Increased water content in irradiated cortex. | No effect on water content |
| Kovacs et al., 2018 | Mouse, n = 12, F, Age; N/A | X-rays; 0, 5 or 20 Gy | Single  PBI | Whole brain | MRI (7T), [18F] fluoro-deoxy-D-glucose- positron emission tomography (FDG-PET) | 2 months | No significant changes in cerebral blood flow compared to baseline values (p > 0.1). | Significantly decreased ADC values in all brain regions compared to baseline values.  Significantly increased glucose uptake all over the brain, mainly in ipsilateral side. | Not studied. |
| Kang et al., 2020 | Mouse, n = 10, F, 6-weeks-old | Neutrons; 0, 1 or 2 Gy | Single  PBI | Hippocampus, Cortex | MRI (9.4T), FDG-PET,  Histochemistry | 8 days | A dose dependant increase in ADC values, and a drop in glucose uptake in both hemispheres compared to baseline values.  A transient decrease in GLUT 1 and GLUT 3 expressions in both hemispheres, mainly in the 2 Gy group. | Not studied. | Not studied. |
| Parente et al., 2020 | Rat, n = 8, M, 7 ± 2-weeks-old | X-rays; 0 or 25 Gy | Single  WBI | Whole brain | FDG-PET, N-^11^C-methyl-4,40-diaminostilbene (^11^C-MeDAS)- PET | 94 days | Not studied. | ^18^F-FDG uptake significantly decreased in all brain regions.  ^11^C-MeDAS uptake dropped, indicating reduced myelin density. | A reduced ^18^F-FDG uptake in amygdala and cortical regions.  ^11^C-MeDAS uptake was not significantly different from control levels. |
| Suckert et al., 2021 | Mouse, n = 80, F, 11-to-13-weeks-old | Protons; 0, 45, 65 or 85 Gy (to C57BL/6 Mice)  OR  0, 40, 60 or 80 Gy, (to C3H/He mice) | Single  PBI | Hippocampus | MRI (1T) | 160 days | No T2W hyperintensities in any dose. | A hyperintense T2 signal followed by hypo-intensities in doses ≥ 65 Gy only. | A hyperintense T2 signal followed by hypo-intensities in doses ≥ 60 Gy only. |
| *EC density and viability* | | | | | | | | | |
| Kamiryo et al., 1996 | Rat, n = 21, M, 250-300g | GKS; 0, 50, 75 or 120 Gy | Single  PBI | Cortex | Histochemistry | 1 to 4 weeks (120 Gy),  1 to 6 months (75 Gy), and 1 to 12 months (50 Gy) | Few dividing ECs after 120 Gy. | No dividing ECs in 50 or 75 Gy. | A few dividing ECs after 75 Gy. |
| Ljubimova et al., 1991 | Rat, n = 182, sex; N/A,  8-to-61-weeks-old | X-rays; 0, 2.5, 5, 10, 25, 40, 60, 100, or 200 Gy | Single  WBI | Cortex, Septum, CC | Histochemistry | 65 weeks | Doses from 5 to 100 Gy induced a 15% decrease in ECs. A decrease of over 15% occurred in a 200 Gy group in all regions. | ≥ 15% EC reduction after a 200 Gy group. | ≥ 15% EC reduction after a 200 Gy group. |
| Collins-Underwood et al., 2008 | Rat EC cultures | Gamma-rays; 0, 1, 2, 5 or 10 Gy) | Single  Cell cultures | Whole brain | Cytochemistry | 1 day | A dose-dependent increase in intracellular reactive oxygen species (ROS) levels. | Not studied. | Not studied. |
| Bouchet et al., 2010 | Rat implanted with 9L gliosarcoma, n = 59, sex; N/A, 10-weeks-old | MRT; 0, 350 (unidirectional) or 700 Gy (bidirectional/crossfired) | Single  PBI | Caudate nucleus | Histochemistry | 45 days | No effect on ECs. | Lost ECs in ipsilateral side after 700 Gy. | Not studied. |
| Mao et al., 2010 | Mouse, n = 175, M, 10-weeks-old | ^56^Fe particles; 0, 0.5, 2 or 4 Gy | Single  WBI | Hippocampus | Histochemistry | 1 year | Not studied. | Not studied. | EC density in DG and CA1 decreased significantly. |
| Cheng et al., 2014 | Rat, n = 96, M, 200-240 g | Gamma-rays; 0 or 60 Gy | Single  PBI | Cortex | Histochemistry | 24 weeks | A significant decrease in CD31+ cells in the irradiated cortex only. | Increased CD31+ cells. | EC numbers in irradiated similar to control numbers |
| Raoufi-Rad et al., 2017 | Mouse ECs | Gamma-rays; 0, 5, 15 or 25 Gy | Single  Cell cultures | Whole brain | Cytochemistry | 5 days | A dose-dependent drop in number of viable cells, and an increase in cellular hypertrophy and nuclei size. | Not studied. | Not studied. |
| *EC structure* |  |  |  |  |  |  |  |  |  |
| Al-samarrai et al., 1975 | Dog, n = 3, M, 1.5-years-old | Neutrons; 0 or 3.75 Gy | Single PBI | Cortex | EM | 1 day | Normal ECs in both hemispheres. | Not studied. | Not studied. |
| Mori et al., 1991 | Mongolian gerbil brain EC cultures | X-rays; 0, 5, 10, 30, and 60 Gy | Single  Cell cultures | Whole brain | Photon-contrast microscopy | 5 days | Nuclear and cytoplasmic swelling at 36 h post-30 and-60 Gy, and at 72 h post-10 Gy.  Cytoplasm vacuolation and giant cell formation at 72 h post-30-and-60 Gy, at 96 h post-10 Gy, and at 130 h post-5 Gy. | Not studied. | Not studied. |
| Cicciarello et al., 1996 | Rat, n = 8, M, 280-320 g | Photons; 0 or 40 Gy (2 Gy/fraction/day, 5 days a week) | Fractions  WBI | Cortex | EM | 15 and 90 days | An intact endothelium. | Not studied. | An intact endothelium. |
| Kamiryo et al., 2001 | Rat, n = 6, M, 250 to 300 g | GKS; 0 or 75 Gy | Single PBI | Cortex | EM | 3.5 months | Not studied. | Not studied. | Swollen ECs in ipsilateral side compared to non-irradiated side. |
| Yuan et al., 2006 | Mouse, n = 46, M, 6-to-7-weeks-old | X-rays; 0 or 40 Gy (2 Gy/day, 5 days/week) | Fractions WBI | Cortex,  Hippocampus | EM | 180 days | Not studied. | Not studied. | Shorter and less dense EC tight junctions, and increased EC vesicular activities. |
| Cheng et al., 2014 | Rat, n = 96, M, 200-240 g | Gamma-rays; 0 or 60 Gy | Single  PBI | Cortex | EM | 24 weeks | No changes. | No changes. | Abnormal ECs from 16 weeks post-IR. |
| *Capillary morphometry* | | | | | | | | | |
| Cavanagh and Hopewell 1972 | Rat, n = 80,  M/F, 3-to-4 months-old | X-rays; 0, 2, 8, 20 or 40 Gy | Single WBI | Subependyma | Histochemistry | 1 year | No effect on vessels. | No effect on vessels. | Slight fibrous vessel wall thickening in doses ≥ 20 Gy. |
| Al-samarrai et al., 1975 | Dog, n = 3, M, 1.5-years-old | Neutrons; 0 or 3.75 Gy | Single PBI | Cortex | EM | 1 day | A slight extravascular space enlargement in both hemispheres. | Not studied. | Not studied. |
| Janzer et al., 1986 | Dog, n = 5, sex; N/A, Adult | Gamma-rays; 0 or 435 Gy (0.05 Gy/h) | Fractions PBI | White matter | Histochemistry | 362 days | Vasogenic oedema in ipsilateral side. | Vasogenic oedema in ipsilateral side. | Vasogenic oedema in ipsilateral side. |
| Ludwig et al., 1987 | Rabbit, n = 13, sex; N/A, 6-to-8-weeks old | Gamma-rays; 0 or 20 Gy | Single  PBI | Cortex, Thalamus, Putamen | Histochemistry | 1 week | Vasogenic oedema. | Not studied. | Not studied. |
|  | Rabbit, n = 12, sex; N/A, 6-to-8-weeks old | Gamma-rays; 0, 48 Gy or 24 Gy (2 Gy/ fraction/day, 5 days a week | Fractions  PBI | Cortex, Thalamus, Putamen | Histochemistry | 4 weeks post-48 Gy or 14 weeks post 24 Gy | Perivascular lymphocytes and plasma cells in putamen. | Not studied. | No effect. |
| Gobbel et al., 1992 | Dog, n = 7, sex; N/A, 1 to 1.5 years old | Gamma-rays; 0 or 20 Gy (0.45 Gy/h) | Single  PBI | Cortex | CT | 6 weeks | Significantly reduced cerebral blood flow and vascular volume in ipsilateral region compared to contralateral side. | Decreased cerebral blood flow and vascular volume in ipsilateral region only. | Not studied. |
| Coderre et al., 1994 | Rat implanted with 9L gliosarcomas to left side, n = 7, sex and age; N/A | BNCT; 0 or 13.4 Gy  OR  X-rays  22.5 Gy to the tumour region | Single  PBI | Cortex, CC, Hippocampus, Striatum | EM | 1 year | Not studied. | Not studied. | Hypervascularisation after x-rays, but not BNCT. |
| Cicciarello et al., 1996 | Rat, n = 8, M, 280-320 g | Photons; 0 or 40 Gy (2 Gy/fraction/day, 5 days a week) | Fractions  WBI | Cortex | Histochemistry, EM. | 15 and 90 days | No changes on vessels. | Not studied. | Collapsed microvessels, and perivascular oedema. |
| Kamiryo et al., 1996 | Rat, n = 21, M, 250-300g | GKS; 0, 50, 75 or 120 Gy | Single  PBI | Cortex | Histochemistry | 1 to 4 weeks (120 Gy),  1 to 6 months (75 Gy), and 1 to 12 months (50 Gy) | Fibrin deposition, capillary thickening, and increased vessel cross-sectional area after 120 Gy. | Increased average vessel cross-sectional area (in 50 and 75 Gy), and vasodilation (in 75 Gy). | Fibrin deposition in capillary wall after 50 and 75 Gy. |
| Serduc et al., 2006 | Mouse, n = 55,  sex: N/A  5-weeks-old | MRT; 0, 312 or 1000 Gy. | Single  PBI | Cortex, Hippocampus | Histochemistry | 3 months | No effect on capillary network. | No effect on capillary network. | No effect on capillary network. |
| Yuan et al., 2006 | Mouse, n = 46, M, 6-to-7-weeks-old | X-rays; 0 or 40 Gy (2 Gy/day, 5 days/week) | Fractionated  WBI | Cortex,  Hippocampus | Two-photon microscopy, Histochemistry | 180 days | Not studied. | No leukocyte adhesion on vessels. | No leukocyte adhesion. |
| Wilson et al., 2009 | Mouse, n = 5, M, 8-to-10-weeks-old | X-rays; 0 or 20 Gy | Single  PBI | Cortex | Photon microscopy, Histochemistry | 2 days | Increased leukocyte adhesion. | Not studied. | Not studied. |
| Bouchet et al., 2010 | Rat implanted with 9L gliosarcoma, n = 59, sex; N/A, 10-weeks-old | MRT; 0, 350 (unidirectional) or 700 Gy (bidirectional/crossfired) | Single  PBI | Caudate nucleus | MRI (4.7T) | 45 days | Blood volume fraction (BVf) not affected. 700 Gy increased ipsilateral vessel size index (VSI) (p < 0.001). | 700 Gy increased BVf, VSI, and a disorganised vascular network in ipsilateral side. | Not studied. |
| Mao et al., 2010 | Mouse, n = 175, M, 10-weeks-old | ^56^Fe particles; 0, 0.5, 2 or 4 Gy | Single  WBI | Hippocampus | Histochemistry | 1 year | Not studied. | Not studied. | Vessels in CA1 region showed an increased diameter, tortuosity, and collapse.  Microvessel length densities in the DG and CA1 decreased significantly. |
| Wu et al., 2010 | Mouse, n = 48, M, 10-weeks-old | X-rays; 0, 10, 12 and 17 Gy | Single  WBI | Whole brain | Histochemistry | 8 months | Not studied. | Microvessel dilation in DG post all doses (10, 12 and 17 Gy). | Vasogenic oedema post all doses. |
| Zawaski et al., 2012 | Rat bearing C6-GFP expressing tumour, n = N/A, M, 6-to-8-weeks-old | Gamma-rays;  0 or 40 Gy (8 Gy/fraction/day) | Fractionated  PBI | Cortex | Histochemistry | 11 days | Increased leukocyte adhesion on vessels in ipsi-and-contralateral normal tissues. | Not studied. | Not studied. |
| Deng et al., 2017 | Mouse, n = 14, M, 8-week-old | X-rays; 0 or 10 Gy | Single  WBI | Cortex, White matter, Hippocampus | Histochemistry | 180 days | No effect on vessels. | No effect on vessels. | Vessel wall thickening. |
| Kovacs et al., 2018 | Mouse, n = 12, F, age; N/A | X-rays; 0, 5 or 20 Gy | Single  PBI | Whole brain | MRI (7T), FDG-PET | 2 months | No significant changes in cerebral blood flow compared to baseline values (p > 0.1). | No effect on vessels. | Not studied. |
| Allen et al., 2020 | Mouse, n = 24, F, 10-weeks-old | Conventional (0.09 Gy/s) or FLASH (6.9 x 10^6^ Gy/s) electron beams; 0 or 25 Gy | Single  WBI | Hippocampus, SVZ | Histochemistry | 1 week | Increased blood vessel volume in the conventional group only. | Not studied. | Not studied. |
|  | Mouse, n = 24, F, 10-weeks-old | Conventional (0.09 Gy/s) or FLASH (6.9 x 10^6^ Gy/s) electron beams; 0 or 10 Gy | Single  WBI | Hippocampus, SVZ | Histochemistry | 1 month | Not studied. | Increased blood vessel volume in the conventional group only. | Not studied. |
| Suckert et al., 2021 | Mouse, n = 80, F, 11-to-13-weeks-old | Protons; 0, 45, 65 or 85 Gy (to C57BL/6 Mouse)  OR  0, 40, 60 or 80 Gy, (to C3H/He mice) | Single  PBI | Hippocampus | MRI (1T) | 160 days | No effect on vessels. | No effect on vessels. | Vasodilatation, perivascular oedema, and chaotic vessel organization in the ipsilateral region in doses ≥ 80 Gy. |
| *Surface proteins* | | | | | | | | | |
| Olschowka et al., 1997 | Mouse, n = 25, M, age; N/A | Gamma-rays; 0, 5, 15, 25 or 35 Gy | Single  PBI | Whole brain | RT-PCR | 6 h | Significantly increased ICAM-1 mRNA levels in ipsilateral sides of 15, 25, and 35 Gy doses. | Not studied. | Not studied. |
|  | Mouse, n = 25, M, age; N/A | Gamma-rays; 0, or 25 Gy | Single  PBI | Hippocampus, Cortex | Histochemistry | 1 week | Increased ICAM-1 and GFAP expression in hippocampi and cortices of both hemispheres, but not in controls. | Not studied. | Not studied. |
| Moore et al., 2004 | Mouse, n = 78, sex; N/A, 6-to-8-weeks old, | Gamma-rays; 0 or 35 Gy | Single  WBI | Cortex | Western blotting, Histochemistry | 1 day | A 1.5-fold increase (p < 0.05) in cyclooxygenase 2+, associated with ICAM-1 and GFAP upregulation. | Not studied. | Not studied. |
| Collins-Underwood et al., 2008 | Rat EC cultures | Gamma-rays; 0, 1, 2, 5 or 10 Gy) | Single  Cell cultures | Whole brain | Western blotting | 1 day | A significant dose-dependent increase in expression of ICAM-1 and PAI-1 compared to controls (p < 0.05). | Not studied. | Not studied. |
| Wilson et al., 2009 | Mouse, n = 5, M, 8-to-10-weeks-old | X-rays; 0 or 20 Gy | Single  PBI | Cortex | Histochemistry | 2 days | Increased TNF and ICAM-1 expression in irradiated brains. | Not studied. | Not studied. |
| Wu et al., 2010 | Mouse, n = 48, M, 10-weeks-old | X-rays; 0, 10 or 35 Gy | Single  WBI | Whole brain | RT-PCR, ELISA | 1 day | Increased mRNA levels and expression of ICAM-1 from 4 h (in 35 Gy) or from 24 h (in 10 Gy). | Not studied. | Not studied. |
|  | Mouse, n = 48, M, 10-weeks-old | X-rays; 0, 10, 12 and 17 Gy | Single  WBI | Whole brain | Histochemistry | 8 months | Not studied. | Increased ICAM-1 immunoreactivity in DG post all doses (10, 12 and 17 Gy). | Increased ICAM-1 immunoreactivity post all doses. |
| Moravan et al., 2011 | Mouse, n = 45, M, 8-to-10-weeks-old | Gamma-rays; 0 or 35 Gy | Single  WBI | Striatum,  White matter | Histochemistry | 1 year | An increased ICAM-1 expression. | An increased ICAM-1 expression. | An increased ICAM-1 expression. |
| Liu et al., 2012 | Murine EC cultures | X-rays; 0 or 25 Gy | Single  Cell cultures | Whole brain | Histochemistry | 3 weeks | Short-term (up to 1 day) upregulation of E-selectin expression.  Significantly increased thrombomodulin expression compared to controls. | Not studied. | Not studied. |
| Zhu et al., 2012 | Rat, n = 30, M/F, 100 ± 20 g | X-rays; 0 or 30 Gy | Single  PBI | Whole brain | MRI (3T) using ICAM1-microsized  particles of iron oxide (MPIO),  Histochemistry | 7 days | An increased MPIO-ICAM-1 binding and ICAM-1 immunoreactivity | Not studied | Not studied |
| Li et al., 2013 | Rat, n = 76, M, 200 - 240 g, | Gamma-rays; 0 or 75 Gy | Single  PBI | Cortex | Histochemistry | 12 weeks | No effect. | Increased CD147 co-localisation with CD31+ ECs. | Not studied. |
| Deng et al., 2017 | Mouse, n = 14, M, 8-weeks-old | X-rays; 0 or 10 Gy | Single  WBI | Cortex, White matter, Hippocampus | Histochemistry | 180 days | Reduced vWF expression. | Reduced vWF expression. | Reduced vWF expression. |
| Raoufi-Rad et al., 2017 | Mouse ECs | Gamma-rays; 0, 5, 15 or 25 Gy | Single,  Cell cultures | Whole brain | Western blotting, RT-PCR, ELISA | 5 days | A significantly increased expression of ICAM-1 (by all doses) and VCAM-1 in ≥ 15 and 25 Gy. | Not studied. | Not studied. |
| Allen et al., 2020 | Mouse, n = 24, F, 10-weeks-old | Conventional (0.09 Gy/s) or FLASH (6.9 x 10^6^ Gy/s) electron beams; 0 or 10 | Single  WBI | Hippocampus, SVZ | Histochemistry | 1 month | A significant drop in endothelial nitric oxide synthase (eNO) expression in the conventional group only. | An increased lectin volume and eNOS/lectin expression in convectional group only. | Not studied. |
| *Junctional proteins* | | | | | | | | | |
| Deng et al., 2017 | Mouse, n = 14, M, 8-weeks-old | X-rays; 0 or 10 Gy | Single  WBI | Cortex, White matter, Hippocampus | Histochemistry | 180 days | Reduced ZO-1 expression. | Reduced ZO-1 expression. | Reduced ZO-1 expression. |
| Allen et al., 2020 | Mouse, n = 24, F, 10-weeks-old | Conventional (0.09 Gy/s) or FLASH (6.9 x 10^6^ Gy/s) electron beams; 0 or 25 Gy | Single  WBI | Hippocampus, SVZ | Histochemistry | 1 week | An increased claudin-5/lectin levels after FLASH IR only.  A significant drop in claudin-5 and occludin expression in the conventional group only. | Not studied. | Not studied. |
|  | Mouse, n = 24, F, 10-weeks-old | Conventional (0.09 Gy/s) or FLASH (6.9 x 10^6^ Gy/s) electron beams; 0 or 10 Gy | Single  WBI | Hippocampus, SVZ | Histochemistry | 1 month | Not studied. | Claudin5/lectin levels decreased in conventional group only.  Occludin/lectin colocalization greatly reduced after both dose-rates. | Not studied. |
| *Gene expression and signalling* | | | | | | | | | |
| Wu et al., 2020 | Rat EC cultures | X-rays; 0 or 20 Gy | Single,  Cell cultures | Whole brain | RNA-Sequencing, Gene ontology and Kyoto Encyclopedia of Genes and Genomes (KEGG) analyses. | 1 day | IR significantly down-regulated 200 transcripts, which are crucial in processes, such as cell cycle, learning, memory, and cell adhesion.  Up-regulated 183 transcripts that are key in lymphocyte activation, apoptosis, chemokine activity, and membrane transporter activities.  Upregulated pathways, like Ca^2+^ and phosphoinositide 3-kinase–Akt signalling. | Not studied. | Not studied. |
| **Extracellular matrix (ECM)** | | | | | | | | | |
| *Structure* | | | | | | | | | |
| Al-samarrai et al., 1975 | Dog, n = 3, M, 1.5-years-old | Neutrons; 0 or 3.75 Gy | Single PBI | Cortex | EM | 1 day | Normal basement membrane (BM) in both hemispheres. | Not studied. | Not studied. |
| Cicciarello et al., 1996 | Rat, n = 8, M, 280-320 g | Photons; 0 or 40 Gy (2 Gy/fraction/day, 5 days a week) | Fractionated  WBI | Cortex | EM | 15 and 90 days | No effect. | Not studied. | Cytoskeletal component loss. |
| Kamiryo et al., 2001 | Rat, n = 6, M, 250-300 g | GKS; 0 or 75 Gy | Single  PBI | Cortex | EM | 3.5 months | Not studied. | Not studied. | BM swelling and thickening in ipsilateral side only. |
| Cheng et al., 2014 | Rat, n = 96, M, 200-240 g | Gamma-rays; 0 or 60 Gy | Single  PBI | Cortex | EM | 24 weeks | No effect. | No effect. | Thickened BM in irradiated side up to 24 weeks post-IR. |
| *Matrix proteins* | | | | | | | | | |
| Serduc et al., 2006 | Mouse, n = 55,  Sex; N/A,  5-weeks-old | MRT; 0, 312 or 1000 Gy. | Single  PBI | Cortex, Hippocampus | Histochemistry | 3 months | No effect on type IV collagen. | No effect on type IV collagen. | No effect on type IV collagen. |
| Ricard et al., 2009 | Mouse, n = 24, F, age; N/A | Monochromatic synchrotron beams (MSB); 0 or 15 Gy | Single  PBI | Cortex | Histochemistry | 6 months | No damage on collagen IV. | No damage on collagen IV. | Not studied. |
| Lee et al., 2012 | Rat, n = 16, M, age; N/A | Gamma-rays; 0 or 10 Gy | Single  WBI | Hippocampus,  Cortex | RT-PCR,  Histochemistry | 1 day | Upregulation of metalloproteinase-2 (MMP-2) and MMP-9 levels by ≥ 3-fold and 1.6-fold, respectively compared to controls.  Increased TIMP-1 mRNA levels, but TIMP-2 not affected.  Increased gelatinolytic activity, and loss of collagen IV in hippocampus. | Not studied. | Not studied. |
|  | Mouse, n = 16, M, age; N/A | Gamma-rays; 0 or 40 Gy (5 Gy/fraction, twice a week | Fractionated  WBI | Hippocampus,  Cortex | RT-PCR,  Histochemistry | 1 day | Upregulation of MMP-2 and MPP9 levels by ≥ 2.1-fold.  Increased gelatinolytic activity, and loss of collagen IV. | Not studied. | Not studied. |
| Li et al., 2013 | Rat, n = 76, M, 200 - 240 g, | Gamma-rays; 0 or 75 Gy | Single  PBI | Cortex | Histochemistry, Western blotting | 12 weeks | No effect on CD147 and MMP-9 protein expression levels. | Increased CD147 and MMP-9 protein levels in ipsilateral side only (p < 0.001). CD147 co-localisation with MMP-2. | Not studied. |
| Politko et al., 2020 | Mouse, n = 54, M, 2-months-old | X-rays; 0 or 7 Gy by a clinical LINAC (6 MeV Elekta Axesse) or a synchrotron research accelerator VEPP-4 | Single  WBI | Cortex, Hippocampus, | Histochemistry, RT-PCR | 3 days | No significant changes in tissue morphology, but decreased cortical brevican (by 3-to-6-fold) and NG2/CSPG4 (10-to-15- fold) expression compared to controls.  VEPP-4 source non-significantly  reduced cortical decorin and brevican levels compared to Elekta Axesse. | Not studied. | Not studied. |
| **Pericytes** | | | | | | | | | |
| *Density and viability* | | | | | | | | | |
| Al-samarrai et al., 1975 | Dog, n = 3, M, 1.5-years-old | Neutrons; 0 or 3.75 Gy | Single  PBI | Cortex | EM | 1 day | Normal pericytes. | Not studied. | Not studied. |
| Bostrom et al., 2014 | Mouse, n = 24, M, 2-weeks-old | Photons; 0 or 8 Gy | Single  WBI | Hippocampus | Histochemistry | 7 weeks | Pericyte coverage in Subgranular zone (SGZ) significantly decreased by 17% compared to controls. | Reduced pericyte coverage in DG only. | Not studied. |
| **Astrocytes and Microglia** | | | | | | | | | |
| *Density and viability* | | | | | | | | | |
| Slatkin et al., 1995 | Rat, n = 36, sex; N/A,  Adult | MRT; 0, 312, 625, 1250, 2500, 5000, or 10,000 Gy | Single  PBI | Cortex | Histochemistry | 1 month | Lost astrocytic nuclei in ≥ 1250 Gy doses. | Lost astrocytic nuclei in ≥ 1250 Gy doses. | Not studied. |
| Sykova et al., 1996 | Rat pups, n = 63, 1-day-old | X-rays; 0, 20 or 40 Gy | Single  PBI | Cortex, White matter | Histochemistry | 20 days | Dose-dependent glial cell pyknosis in both sides. | Not studied. | Not studied. |
| Noel et al., 1997 | Rat astrocyte cultures | X-rays; 0, 1, 2, 5, or 10 Gy | Single  Cell cultures | Cortex | RT-PCR,  Cytochemistry | 2 days | A significant reduction in mRNA levels of basic fibroblast growth factor after all doses, but no significant changes in cell survival. | Not studied. | Not studied. |
| Tada et al., 1999 | Rat, n = 24, M, 8-to-10-weeks-old | X-rays; 0, 2, 5, 7.5, 10, or 15 Gy | Single  WBI | Subependyma | Histochemistry | 180 days | No effect on astrocytes. | Not studied. | Dose dependent loss of astrocytes in doses ≥ 5 Gy. |
| Serduc et al., 2006 | Mouse, n = 55, sex; N/A, 5-weeks-old | MRT; 0, 312 or 1000 Gy. | Single  PBI | Cortex, Hippocampus | Histochemistry | 1 month | Significant loss of glial cells in the ipsilateral DG. | Significant loss of glial cells in the ipsilateral DG. | Not studied. |
| Hua et al., 2012 | Rat, n = 80, M, 8-and-28-months-old | Gamma-rays; 0 or 10 Gy | Single  WBI | Hippocampus | Histochemistry | 10 weeks | Microglial density decreased by 20% to 25% in both ages. | Microglial density similar to control levels in both ages. | Not studied. |
| Blomstrand et al., 2014 | Rat, n = 15, M, 9-days and 6-months-old | Photons; 0 or 6 Gy | Single  WBI | Hippocampus | Histochemistry | 4 weeks | Microglia density increased by 12% in juvenile, and decreased by 35% in adults. | Not studied. | Not studied. |
| Politko et al., 2021 | Mouse, n = 36, M, 2-months-old | X-rays; 0 or 21 Gy (7Gy/fraction/ day) | Fractionated  WBI | Cortex | RT-PCR,  Histochemistry, Dot-blot analysis | 3 days | Short-term (up to 2 days) upregulation of brevan, decorin and CD44, and downregulation of versican in cortex. In subcortex, no tissue changes, but reduced brevican staining.  A decrease in overall content of heparan sulphate chains (p < 0.05) and a non-significant reduction in chondroitin sulphate content in the cortex. | Not studied. | Not studied. |
|  | Non-irradiated or irradiated normal glia cells cultured with U87 cancer cells | X-rays; 0 or 21 Gy (7Gy/fraction/ day) | Fractionated  Cell cultures | Cortex | Cytochemistry, Confocal microscopy | 3 days | Irradiated glial cells had a doubling time of 28–29 days versus 4–5 days for no-IR glial cultures.  Number of U87 cells significantly increased in presence of irradiated normal glial cells, but U87 adhesion and proliferation was restricted in presence of non-irradiated glia cells. | Not studied. | Not studied. |
| *Cell structure* | | | | | | | | | |
| Al-samarrai et al., 1975 | Dog, n = 3, M, 1.5-years-old | Neutrons; 0 or 3.75 Gy | Single  PBI | Cortex | EM | 1 day | Normal glial elements. | Not studied. | Not studied. |
| Kamiryo et al., 2001 | Rat, n = 6, M, 250-300 g | GKS; 0 or 75 Gy | Single  PBI | Cortex | EM | 3.5 months | Not studied. | Not studied. | Increased glial filaments. |
| Cheng et al., 2014 | Rat, n = 96, M, 200-240 g | Gamma-rays; 0 or 60 Gy | Single  PBI | Cortex | EM | 24 weeks | No effect. | No effect. | Astrocytic feet swelling in irradiated side up to 24 weeks post-IR. |
| *Protein expression* | | | | | | | | | |
| Janzer et al., 1986 | Dog, n = 5, sex; N/A, Adult | Gamma rays; 0 or 435 Gy (0.05 Gy/h) | Fractionated  PBI | White matter | Histochemistry | 362 days | Increased GFAP expression in ipsilateral side. | Increased GFAP expression in ipsilateral side. | Increased GFAP expression  in both hemispheres. |
| Ludwig et al., 1987 | Rabbit, n = 13, sex; N/A, 6-to-8-weeks old | Gamma-rays; 0 or 20 Gy | Single  PBI | Cortex, Thalamus, Putamen | Histochemistry | 1 week | Astrocytic reactivity. | Not studied. | Not studied. |
|  | Rabbit, n = 12, sex; N/A, 6-to-8-weeks old | Gamma-rays; 0 or 48 Gy or 24 Gy (2 Gy/ fraction/day, 5 days a week | Fractionated  PBI | Cortex, Thalamus, Putamen | Histochemistry | 4 weeks post-48 Gy or 14 weeks post 24 Gy | Mild microglial and astrocytic reactions in cortex. | Not studied. | Minimal astrocytic reactivity. |
| Chiang et al., 1991 | Mouse astrocyte & microglial cultures | Gamma-rays; 0, 2, 4 or 8 Gy | Single  Cell cultures | Whole brain | Cytochemistry | 1 day | No significant increases in TNF-a production by both glial cells. | Not studied. | Not studied. |
| Sawaya et al., 1994 | Rat astrocyte cultures | X-rays; 0 or 10 Gy | Single  Cell cultures | Cortex | Enzyme zymography, Enzyme Linked Immunosorbent Assay (ELISA) | 2 days | No significant changes in levels and activity of extracellular tissue plasminogen activators (tPA) and intracellular type-IV collagenase.  Significant increases in amount (>5-fold) and activity (3-to-4-fold) of intracellular tPA.  Increased extracellular levels (> 7-fold higher) and activity (5- to 6-fold higher) of collagenase IV. | Not studied. | Not studied. |
| Sakuma et al., 1995 | Rat astrocyte cultures | X-rays; 0 or 10 Gy | Single  Cell cultures | Whole brain | RT-PCR,  Northern blotting | 1 day | Increased protein tyrosine kinase receptor 3 (PTK-3) expression after IR. | Not studied. | Not studied. |
| Kamiryo et al., 1996 | Rat, n = 21, M, 250-300g | GKS; 0, 50, 75 or 120 Gy | Single  PBI | Cortex | Histochemistry | 1 to 4 weeks (120 Gy),  1 to 12 months (50 and 75 Gy) | Increased GFAP expression after 120 Gy. | Increased GFAP expression after 75 Gy. | Increased GFAP expression after 50 and 75 Gy. |
| Sykova et al., 1996 | Rat pups, n = 63, sex; N/A, 1-day-old | X-rays; 0, 20 or 40 Gy | Single  PBI | Cortex, White matter | Histochemistry | 20 days | Increased GFAP expression in both sides, but predominant in ipsilateral region of 40 Gy. | Not studied. | Not studied. |
| Olschowka et al., 1997 | Mouse, n = 25, M, age; N/A | Gamma-rays; 0 or 25 Gy | Single  PBI | Hippocampus, Cortex | Histochemistry | 1 week | A significant upregulation of GFAP in both hemispheres, but not in controls. | Not studied. | Not studied. |
| Kim et al., 1999 | Rat with 9L gliosarcoma in left cerebrum, n = N/A, M, 200-240g | Gamma-rays; 0, 25 or 35 Gy | Single  PBI | Whole brain | Histochemistry | 180 days | Not studied. | Not studied. | A dose dependent increase in GFAP expression in ipsilateral normal tissues. |
| Shi et al., 2002 | Rat, n = 15, M, 200-220 g | X-rays; 0 or 20 Gy | Single  WBI | Ependyma, Subependyma | Histochemistry | 4 weeks | Increased GFAP immunoreactivity. | Not studied. | Not studied. |
| Moore et al., 2004 | Mouse, n = 78, sex; N/A, 6-to-8-weeks old | Gamma-rays; 0 or 35 Gy | Single  WBI | Cortex | Histochemistry | 1 day | Increased GFAP upregulation. | Not studied. | Not studied. |
| Yuan et al., 2006 | Mouse, n = 46, M, 6-to-7-weeks-old | X-rays; 0 or 40 Gy (2 Gy/day, 5 days/week) | Fractionated  WBI | Cortex,  Hippocampus | Histochemistry | 180 days | Not studied. | Increased GFAP expression, mainly in the cortex. | Increased GFAP expression, mainly in the cortex. |
| Ricard et al., 2009 | Mouse, n = 24, F, age; N/A | Monochromatic synchrotron beams (MSB); 0 or 15 Gy | Single  PBI | Cortex | Histochemistry | 6 months | No gliosis. | No gliosis. | No gliosis. |
| Wilson et al., 2009 | Mouse, n = 5, M, 8-to-10-weeks-old | X-rays; 0 or 20 Gy | Single  PBI | Cortex | Histochemistry | 2 days | Increased GFAP expression. | Not studied. | Not studied. |
| Moravan et al., 2011 | Mouse, n = 45, M, 8-to-10-weeks-old | Gamma-rays; 0 or 35 Gy | Single  WBI | Striatum,  White matter | Histochemistry | 1 year | Increased GFAP expression. | Increased GFAP expression. | Increased GFAP expression. |
| Schindler et al., 2011 | Rat, n = 18, M, 8-months-old | Gamma-rays; 0 or 10 Gy | Single  PBI | Hippocampus | Histochemistry | 70 days | An increased IBA1+, and BrdU/IBA-1+ cells in ipsilateral regions only. | Reduced number of IBA1+ cells compared to controls. | Not studied. |
| Zawaski et al., 2012 | Rat bearing C6-GFP expressing tumour, n = N/A, M, 6-to-8-weeks-old | Gamma-rays; 0 or 40 Gy (8 Gy/fraction/day) | Fractionated  PBI | Cortex | Histochemistry | 11 days | Increased GFAP expression in peritumoral normal tissues. | Not studied. | Not studied. |
| Li et al., 2013 | Rat, n = 76, M, 200 - 240 g, | Gamma-rays; 0 or 75 Gy | Single  PBI | Cortex | Histochemistry | 12 weeks | No effect. | Increased CD147 co-localisation with GFAP+ astrocytes. | Not studied. |
| Deng et al., 2017 | Mouse, n = 14, M, 8-weeks-old | X-rays; 0 or 10 Gy | Single  WBI | Cortex, White matter, Hippocampus | Histochemistry | 180 days | Increased expression of GFAP, and GFAP/VEGF. | Increased expression of GFAP, and GFAP/VEGF. | Increased expression of GFAP, and GFAP/VEGF. |
| Prezado et al., 2017 | Rat, n = 23, sex; N/A,  1.5-months-old | Conventional/BB protons (PRT)  Or proton minibeam RT (pMBRT); 0 or 25 Gy | Single  WBI | Whole brain | Histochemistry | 6 months | No pathologies in any animals. | Not studied. | Only PRT increased GFAP and IBA-1+ cells in the hippocampus, hypothalamus, periaqueductal gray, basal forebrain and brainstem. |
| Montay-Gruel et al., 2020 | Mouse, n = 12, F, 3-to-4-months-old | Conventional (0.1 Gy/s) or FLASH (5.6 × 10^6^ Gy/ s) electron beams; 0 or 10 Gy | Single  WBI | Hippocampus | Histochemistry | 1 month | Not studied. | Increased GFAP expression, and elevated expression of IBA1+/ C1q, TLR4, and TLR4/GFAP in conventional IR group only.  A significant rise in total C3 and in GFAP+/C3 staining after IR.  An increased C1q expression especially after FLASH IR. | Not studied. |
| Suckert et al., 2021 | Mouse, n = 80, F, 11-to-13-weeks-old | Protons  0, 45, 65 or 85 Gy (to C57BL/6 mice)  OR  0, 40, 60 or 80 Gy, (to C3H/He mice) | Single  PBI | Hippocampus | Histochemistry | 160 days | No effect. | No effect. | Increased IBA-1+ and GFAP+ cells in ipsilateral hippocampus in doses ≥ 80 Gy. |
| **Oligodendrocytes** | | | | | | | | | |
| *Cell structure, density + viability* | | | | | | | | | |
| Al-samarrai et al., 1975 | Dog, n = 3, M, 1.5-years-old | Neutrons; 0 or 3.75 Gy | Single  PBI | Cortex | EM | 1 day | Normal oligodendrocytes. | Not studied. | Not studied. |
| Panagiotakos et al., 2007 | Rat, n = 28, F, 3-months-old | X-rays; 0 or 25 Gy | Single  WBI | SVZ, Cortex, CC | Histochemistry | 15 months | Significant loss of oligodendrocytes in irradiated brains. | Not studied. | Significant loss of oligodendrocytes in irradiated brains. |
| Shi et al., 2009 | Rat, n = 31, M, 12-months-old | Gamma-rays; 0 or 45 Gy (5 Gy/fraction, twice a week) | Fractionated  WBI | CC, Anterior commissure (AC), Cortex | Histochemistry | 12 months | Not studied. | Not studied. | Oligodendrocyte numbers not affected (p > 0.05). |
| Fu et al., 2017 | Mouse, n = 72, M, 3, 4, and 8-weeks-old | Gamma-rays; 0 or 25 Gy | Singe  WBI | Cortex, White matter | Histochemistry | 5 months | Loss of oligodendrocytes (up to 2 weeks) in cortex and CC of 3 and 4-weeks-old irradiated group only. | No effect. | No effect. |
| **Neurons** | | | | | | | | | |
| *Cell density and viability* | | | | | | | | | |
| Slatkin et al., 1995 | Rat, n = 36, sex; N/A,  Adult | MRT; 0, 312, 625, 1250, 2500, 5000, or 10,000 Gy | Single  PBI | Cortex | Histochemistry | 1 month | Loss of neuronal nuclei in doses ≥ 1250 Gy. | Loss of neuronal nuclei in doses ≥ 1250 Gy. | Not studied. |
| Kamiryo et al., 1996 | Rat, n = 21, M, 250-300g | GKS; 0, 50, 75 or 120 Gy | Single  PBI | Cortex | Histochemistry | 1 to 4 weeks (120 Gy),  1 to 6 months (75 Gy), and 1 to 12 months (50 Gy) | Neuronal loss after 120 Gy. | No effect. | Neuronal loss in 75 Gy. |
| Tada et al., 1999 | Rat, n = 24, M, 8-to-10 weeks | X-rays; 0, 2, 5, 7.5, 10, or 15 Gy | Single  WBI | Subependyma | Histochemistry | 180 days | A 98.1% reduction in neurons compared to controls. | No effect. | Loss of neurons in doses ≥ 5 Gy. |
| Shi et al., 2002 | Rat, n = 15, M, 200-220 g | X-rays; 0 or 20 Gy | Single  WBI | Ependyma, Subependyma | Histochemistry | 4 weeks | Upregulated nestin+ cells, and reduced nestin/Ki-67 cells. | Not studied. | Not studied. |
| Raber et al., 2004 | Mouse, n = 28, M, 2-months-old | X-rays; 0 or 10 Gy | Single  WBI | Hippocampus, White matter | Histochemistry | 3 months | Not studied. | Not studied. | Number of neurons in DG reduced by ~ 90% compared to controls. |
| Fukuda et al., 2005 | Rat pups, n = 10, sex; N/A, 9 (P9) and 23 (P23)-days-old | Photons; 0 or 8 Gy | Single  PBI | SVZ, Hippocampus, Striatum, CC, Cortex | Histochemistry | 10 weeks | Number of nestin+ and doublecortin+ cells in P9 and P23 significantly dropped by ≥ 28 % in SVZ and DG of both hemispheres.  A significant loss of NeuN+ cells (in P9) and in both hemispheres, but not in controls. | Number of nestin+ and doublecortin+ cells in P9 and P23 significantly dropped by ≥ 28% in SVZ and DG of both brain sides.  NeuN+ cells decreased in both sides (P9) or ipsilateral side only (P23). | Not studied. |
| Serduc et al., 2006 | Mouse, n = 55, sex; N/A, 5-weeks-old | MRT; 0, 312 or 1000 Gy. | Single  PBI | Cortex, Hippocampus | Histochemistry | 1 month | Lost neuronal cells in the ipsilateral DG, but not in the contralateral side. | Lost neuronal cells in the ipsilateral DG, but not in the contralateral side. | Not studied. |
| Panagiotakos et al., 2007 | Rat, n = 28, F, 3-months-old | X-rays; 0 or 25 Gy | Single  WBI | SVZ, Cortex, CC | Histochemistry | 15 months | Significant loss of doublecortin+ neuroblasts in SVZ, cortex and CC of irradiated brains. | Not studied. | Significant loss of doublecortin+ neuroblasts in SVZ, cortex and CC of irradiated brains. |
| Ricard et al., 2009 | Mouse, n = 24, F, age; N/A | Monochromatic synchrotron beams (MSB); 0 or 15 Gy | Single  PBI | Cortex | Histochemistry | 6 months | No effect on neurons. | No effect on neurons. | Neither neuronal maturation induced. |
| Wu et al., 2010 | Mouse, n = 48, M, 10-weeks-old | X-rays; 0, 10, 12 and 17 Gy | Single  WBI | Whole brain | Histochemistry | 8 months | Not studied. | No effect. | Reduced neuronal proliferation in DG. |
| Park et al., 2011 | Mouse bearing tumours, n = 23, F, 6-to-8-weeks-old | X-rays; 0 or 14 Gy | Single  WBI | Hippocampus, Cortex | Histochemistry | 1 day | Neuronal cell death in ipsilateral normal tissue. | Not studied. | Not studied. |
| Schindler et al., 2011 | Rat, n = 18, M, 8-months-old | Gamma-rays; 0 or 10 Gy | Single  PBI | Hippocampus | Histochemistry | 70 days | Reduced neuronal proliferation. | Reduced neuronal proliferation. | Not studied. |
| Gazdzinski et al., 2012 | Mouse, n = 35, F, 2.5-weeks-old | Gamma-rays; 0 or 7 Gy | Single  WBI | Whole brain | Histochemistry | 6.5 weeks | A loss of doublecortin+ cells in SGZ. | Loss of doublecortin+ cells in SGZ. | Not studied. |
| Ji et al., 2014 | Rat, n = 12, sex; N/A, 150-200 g, | Electrons; 0 or 30 Gy | Single  WBI | Hippocampus | Histochemistry | 30 days | Number of BrdU/NeuN+ cells reduced by 67%. | BrdU/ NeuN+ cells were almost absent. | Not studied. |
| Beera et al., 2018 | Mouse, n = 15, sex; N/A, 16-days-old | X-rays; 0 or 8 Gy | Single  WBI or PBI | CC, Hippocampus, SVZ | Histochemistry | 47 days | Not Studied. | Loss of doublecortin+ cells in the SVZ, especially after WBI. | Not studied. |
| *Protein expression and cell signalling* | | | | | | | | | |
| Janzer et al., 1986 | Dog, n = 5, sex; N/A, Adult | Gamma rays; 0 or 435 Gy (0.05 Gy/h) | Fractionated  PBI | White matter | Histochemistry | 362 days | No effect. | Slight neuron-specific enolase reactivity in ipsilateral side. | Increased neuron-specific enolase reactivity in both hemispheres. |
| Ji et al., 2014 | Rat, n = 12, sex; N/A, 150-200 g, | Electrons; 0 or 30 Gy | Single  WBI | Hippocampus | RT-PCR, Western blotting | 30 days | BDNF and TrkB levels greatly reduced, and H3 acetylation reduced by 30%. | BDNF and TrkB levels greatly reduced, and H3 acetylation dropped by 61%. | Not studied. |
| Pius-Sadowska et al., 2016 | Mouse, n = 30, F, 6-to-8-weeks-old | Gamma-rays; 0 or 10 Gy | Single  WBI | Hippocampus, SVZ | RT-PCR, Western blotting, Histochemistry | 7 days | A transient upregulation of neurotrophins and their receptors in the SGZ and SVZ compared to controls.  Activated Akt and MAPK signalling. | Not studied. | Not studied. |
| *Neural microstructure* | | | | | | | | | |
| Cavanagh and Hopewell 1972 | Rat, n = 80,  M/F, 3-to-4 months old | X-rays; 0, 2, 8, 20 or 40 Gy | Single  WBI | Subependyma | Histochemistry | 1 year | No effect. | No effect. | Necrosis in doses ≥ 20 Gy. |
| Jacobs et al., 1986 | Rat, n = 80, sex; N/A, 4-days-old | Gamma-rays; 0, 5, 15, 20, or 25 Gy | Single  WBI | Whole brain | [^35^S] sulphate incorporation assay | 22 days | [35S] sulphate  incorporation in irradiated animals dropped by ≥ 30% in all doses. | Not studied. | Not studied. |
|  | Rat brain reaggregate cultures | Gamma-rays; 0, 2.5, 5, 10, or 15 Gy | Single  Cell cultures | Whole brain | [^35^S] sulphate incorporation assay | 22 days | Significantly reduced incorporation in ≥ 10Gy doses. | Not studied. | Not studied. |
| Janzer et al., 1986 | Dog, n = 5, sex; N/A, Adult | Gamma rays; 0 or 435 Gy (0.05 Gy/h) | Fractionated  PBI | White matter | Histochemistry | 362 days | Demyelination in ipsilateral side. | Demyelination in ipsilateral side. | Demyelination in ipsilateral side. |
| Ludwig et al., 1987 | Rabbit, n = 13, sex; N/A, 6-to-8-weeks old | Gamma-rays; 0 or 20 Gy | Single  PBI | Cortex, Thalamus, Putamen | Histochemistry | 1 week | Myelin swelling. | Not studied. | Not studied. |
|  | Rabbit, n = 12, sex; N/A, 6-to-8-weeks old | Gamma-rays; 0, 48 Gy or 24 Gy (2 Gy/ fraction/day, 5 days a week | Fractionated  PBI | Cortex, Thalamus, Putamen | Histochemistry | 4 weeks post-48 Gy or 14 weeks post 24 Gy | Minor myelin swelling and beading in thalamus. | Not studied. | No effect seen. |
| Ljubimova et al., 1991 | Rat, n = 182, sex; N/A,  8-to-61 weeks-old | X-rays; 0, 2.5, 5, 10, 25, 40, 60, 100, or 200 Gy | Single  WBI | Cortex, Septum, CC | EM, Histochemistry | 65 weeks | No effect. | No effect. | Extensive necrosis, mainly in the CC and septal area, from week 39 in doses ≥ 40 Gy. |
| Fike et al., 1995 | Dog, n = N/A, M, 1-1.5-years-old. | Gamma-rays; 0 or 20 Gy (0.45 Gy/h) | Single  PBI | White matter | Histochemistry | 8 weeks | Necrosis with inflammatory cells, mainly leukocytes, in the ipsilateral side. | No necrosis, but increased mononuclear cells (macrophages or reactive microglia) in ipsilateral region. | Not studied. |
| Cicciarello et al., 1996 | Rat, n = 8, M, 280-320 g | Photons;  0 or 40 Gy (2 Gy/fraction/day, 5 days a week) | Fractionated  WBI | Cortex | Histochemistry, EM | 15 and 90 days | No structural changes in neuronal bodies. | Not studied. | Myelin sheath swelling, mitochondrial alterations and irregular bodies in the neuropil, increased density in synaptic contacts, rarefaction of the presynaptic vesicles |
| Kamiryo et al., 1996 | Rat, n = 21, M, 250-300g | GKS; 0, 100, 120, 140, 160, 180, or 200 Gy | Single  PBI | Cortex | Histochemistry | 1 month | Not studied. | Necrosis in all doses. | Not studied. |
|  | Rat, n = 21, M, 250-300g | GKS; 0, 50, 75 or 120 Gy | Single  PBI | Cortex | Histochemistry | 1 to 4 weeks (120 Gy),  1 to 6 months (75 Gy), and 1 to 12 months (50 Gy) | Necrosis, neuron and myelin loss after 120 Gy. | No effect. | Necrosis, lymphocytic infiltration, neurons and myelin loss in 75 Gy. |
| Sykova et al., 1996 | Rat pups, n = 63, sex; N/A, 1-day-old | X-rays;  0, 20 or 40 Gy | Single  PBI | Cortex, White matter | Histochemistry, Real-time iontophoresis | 20 days | Neuronal cell pyknosis, necrosis, macrophage infiltration, increased extracellular space volume, tortuosity and non-specific uptake in both sides, but predominant in ipsilateral region of 40 Gy. | Not studied. | Not studied. |
| Kim et al., 1999 | Rat with 9L gliosarcoma in left cerebrum, n = N/A, M, 200-240g | Gamma-rays; 0, 25 or 35 Gy  OR  14 Gy x 3 or 17.5 Gy x 2 | Single  PBI  OR  Fractionated  PBI | Whole brain | Histochemistry | 180 days | Not studied. | Not studied. | Severe necrosis, myelin fragmentation in ipsilateral normal tissue region of 35 Gy. Mild effects in 25 Gy.  Less neural tissue damage in fractionated regimens than in single doses in both hemispheres. |
| Daigle et al., 2001 | Mouse, n = 30, sex; N/A, 6-to-8-weeks-old | X-rays; 0 or 25 Gy | Single  PBI | Hippocampus | Histochemistry, ELISA | 5.5 months | No effect. | No effect. | Neuronal degeneration and a 20% decrease in total myelin basic protein in irradiated animals. |
| Kamiryo et al., 2001 | Rat, n = 6, M, 250 to 300 g | GKS; 0 or 75 Gy | Single PBI | Cortex | EM | 3.5 months | Not studied. | Not studied. | A normal neuropil ultrastructure. |
| Fukuda et al., 2005 | Rat pups, n = 10, sex; N/A, 9 (P9) or 23 (P23)-days-old | Photons; 0 or 8 Gy | Single  PBI | SVZ Hippocampus, Striatum, CC, Cortex | Histochemistry | 10 weeks | A significant loss of MBP in P9 and P23, in both hemispheres, but not in controls. | MBP decreased in both sides (P9) or ipsilateral side only (P23). | Not studied. |
| Ernst-Stecken et al., 2007 | Rat, n = 9, M, Adult | X-rays; 0, 20, 30 or 40 Gy (10 Gy/ fraction/week) | Fractionated  PBI | Hippocampus, SVZ | Histochemistry | 16 weeks | Not studied. | Necrosis in ipsilateral side of 30 Gy group or both brain sides after 40 Gy in SVZ. | Necrosis in ipsilateral side of 20 and 30 Gy or both brain sides after 40 Gy in SVZ. |
| Panagiotakos et al., 2007 | Rat, n = 28, F, 3-months-old | X-rays; 0 or 25 Gy | Single  WBI | SVZ, Cortex, CC | EM, Histochemistry | 15 months | No effect. | Not studied. | Demyelination and necrosis from 9 to 15 months. |
| Shi et al., 2009 | Rat, n = 31, M, 12-months-old | Gamma-rays; 0 or 45 Gy (5 Gy/fraction, twice a week) | Fractionated  WBI | CC, Anterior commissure (AC), Cortex | Histochemistry, EM | 12 months | Not studied. | Not studied. | No demyelination nor necrosis in CC, AC or cortex (p = 0.58). |
| Wu et al., 2010 | Mouse, n = 48, M, 10-weeks-old | X-rays; 0, 10, 12 and 17 Gy | Single  WBI | Whole brain | Histochemistry | 8 months | Not studied. | No effect. | Demyelination and axonal swelling in white matter of irradiated brains. |
| Moravan et al., 2011 | Mouse, n = 45, M, 8-to-10-weeks-old | Gamma-rays; 0 or 35 Gy | Single  WBI | Striatum,  White matter | Histochemistry | 1 year | No necrosis, but an increased 7/4+ cells in tissue. | No necrosis, but an increased 7/4+, CD3 and CD11c+ cells in tissue. | No necrosis, but an increased 7/4+, CD3 and CD11c+ cells in tissue. |
| Gazdzinski et al., 2012 | Mouse, n = 35, F, 2.5-weeks-old | Gamma-rays; 0 or 7 Gy | Single  WBI | Whole brain | Histochemistry | 6.5 weeks | Loss of myelin basic protein (MBP) in white matter. | Loss MBP in white matter. | Not studied. |
| Bostrom et al., 2014 | Mouse, n = 24, M, 2-weeks-old | Photons; 0 or 8 Gy | Single  WBI | Hippocampus | Histochemistry | 7 weeks | Transient (up to 6 h post-IR) increase in distance between vessels and the nearest doublecortin+ cells and Ki-67+ cells. | No effect. | Not studied. |
| Cheng et al., 2014 | Rat, n = 96, M, 200-240 g | Gamma-rays; 0 or 60 Gy | Single  PBI | Cortex | EM | 24 weeks | No effect. | No effect. | Increased chromatin condensation in neurons in irradiated side up to 24 weeks post-IR. |
| Serduc et al., 2014 | Rat, n = 48,  sex and age; N/A | Micro- or Minibeams; 0, 150, 280, or 500 Gy | Single  PBI | White matter | MRI (9.4T) | 2 months | Not studied | Demyelination shown by: a dose-dependant drop in functional anisotropy values and a significantly increased axial and radial diffusivity values compared to controls (p < 0.05). | Not studied. |
| Deng et al., 2017 | Mouse, n = 14, M, 8-weeks-old | X-rays; 0 or 10 Gy | Single  WBI | Cortex, White matter, Hippocampus | Histochemistry | 180 days | No effect. | No effect. | Hippocampal necrosis. |
| Fu et al., 2017 | Mouse, n = 72, M, 3, 4, and 8-weeks-old | Gamma-rays; 0 or 25 Gy | Singe  WBI | Cortex, White matter | Histochemistry, Western blotting | 5 months | Demyelination in all ages, but most severe in 8-weeks-old group. | Demyelination in the 8-weeks-old group only. | Demyelination in the 8-weeks-old group only. |
| Prezado et al., 2017 | Rat, n = 23, sex; N/A,  1.5-months-old | Conventional/BB protons (PRT)  Or proton minibeam RT (pMBRT); 0 or 25 Gy | Single  WBI | Whole brain | Histochemistry | 6 months | No pathologies in any animals. | Not studied. | Only PRT induced neuropil necrosis, demyelination, and mineralisation in the hippocampus, hypothalamus, periaqueductal gray, basal forebrain and brainstem. |
| Beera et al., 2018 | Mouse, n = N/A, sex; N/A,16-days-old | X-rays;  0 or 8 Gy | Single  WBI or PBI | CC, Hippocampus, SVZ | Histochemistry | 47 days | Not studied. | A significant loss of MBP in CC after WBI, but not in  focal or control groups. | Not studied. |
| Suckert et al., 2021 | Mouse, n = 80, F, 11-to-13-weeks-old | Protons; 0, 45, 65 or 85 Gy (to C57BL/6 mice)  OR  0, 40, 60 or 80 Gy, (to C3H/He mice) | Single  PBI | Hippocampus | Histochemistry | 160 days | No effect. | Minor tissue changes in 60/65 Gy doses. | Necrosis in ipsilateral regions in doses ≥ 80 Gy. |
| **Changes non-specific to any NVU component** | | | | | | | | | |
| Cavanagh and Hopewell 1972 | Rat, n = 80,  M/F, 3-to-4 months-old | X-rays; 0, 2, 8, 20 or 40 Gy | Single  WBI | Subependyma | Histochemistry | 1 year | A dose-dependent nuclei pyknosis and a reduced number of cells undergoing mitosis. | Reduced number of cells undergoing mitosis in doses ≥ 20 Gy. | Chromosome fragments in doses ≥ 8 Gy, and cell loss in ≥ 20 Gy. |
| Chauser et al., 1977 | Rat, n = 28, F, 8-weeks-old | X-rays; 0, 1, 2.5, 5, 10, 15, or 30 Gy  OR  Neutrons; 0.5, 1.15, 3, 5, or 8 Gy | Single  WBI | Subependyma | Histochemistry | 6 months | Transient cell loss in ≤ 10 Gy of x-rays. Significant cell loss in ≥ 15 Gy of x-rays and in ≥ 1.15 Gy of neutron-irradiation. | Significant cell loss in ≥ 15 Gy of x-ray and in ≥ 1.15 Gy of neutron-irradiation. | Significant cell loss in ≥ 15 Gy of x-rays and ≥ 1.15 Gy of neutron-irradiation. |
| Bellinzonna et al., 1996 | Rat, n = 12, F, 2-to-3-months-old | X-rays; 0, 5 or 30 Gy | Single  WBI | Subependyma, Cortex, CC, Striatum | Histochemistry | 2 days | A transient dose-dependent increase in apoptosis (up to 6 h post both doses) in the subependyma and CC compared to controls. | Not studied. | Not studied. |
| Shinohara et al., 1997 | Rat, n = 12, M, 8-to-10 weeks | X-rays; 0, 1.4 or 15 Gy | Single  WBI | Whole brain | Histochemistry | 2 days | Both doses greatly induced apoptosis in the subependyma and CC, but no effect in controls. | Not studied. | Not studied. |
| Tada et al., 1999 | Rat, n = 24, M, 8-to-10-weeks-old | X-rays; 0, 2, 5, 7.5, 10, or 15 Gy | Single  WBI | Subependyma | Histochemistry | 180 days | IR induced a 95.5% decrease in dividing cells compared to controls, but no apoptosis detected. | Reduced number of dividing cells, but no IR-induced cell death. | No unspecific effects. |
| Daigle et al., 2001 | Mouse, n = 30, sex; N/A, 6-to-8-weeks-old | X-rays; 0 or 25 Gy | Single  PBI | Hippocampus | Histochemistry | 5.5 months | Short-term increase in cell death, mainly in DG. | A non-significant increase in cell proliferation in DG. | No unspecific effects. |
| Fukuda et al., 2005 | Rat pups, n = 10, sex; N/A, 9 (P9) or 23 (P23)-days-old | Photons; 0 or 8 Gy | Single  PBI | SVZ, Hippocampus, Striatum, CC, Cortex | Histochemistry | 10 weeks | Increased activation of caspase-3, p53, and nitrotyrosine in SVZ and DG of both hemispheres in P9 compared to P23 and control groups. | No unspecific effects. | Not studied. |
| Serduc et al., 2006 | Mouse, n = 55, sex; N/A, 5-weeks-old | MRT; 0, 312 or 1000 Gy. | Single  PBI | Cortex, Hippocampus | Histochemistry | 3 months | Increased pyknotic nuclei in the ipsilateral DG only. | No unspecific effects. | No unspecific effects. |
| Panagiotakos et al., 2007 | Rat, n = 28, F, 3-months-old | X-rays; 0 or 25 Gy | Single  WBI | SVZ, Cortex, CC | Histochemistry | 15 months | Significant loss of proliferating cells in the SVZ, cortex and CC of irradiated brains. | Not studied. | Significant loss of proliferating cells in the SVZ, cortex and CC of irradiated brains. |
| Bouchet et al., 2010 | Rat implanted with 9L gliosarcoma, n = 59, sex; N/A, 10-weeks-old | MRT; 0, 350 (unidirectional) or 700 Gy (bidirectional/crossfired) | Single  PBI | Caudate nucleus | Western blotting | 45 days | Increased VEGF expression by both doses. | VEGF amounts in ipsilateral side was > 2.5-times higher than that of contralateral side after 350 Gy. | Not studied. |
| Schindler et al., 2011 | Rat, n = 18, M, 8-months-old | Gamma-rays; 0 or 10 Gy | Single  PBI | Hippocampus | Histochemistry | 70 days | A 10-fold increase in pyknotic nuclei in the DG. An increased γ-H2AX signal (DNA damage) in ipsilateral regions only. | No unspecific effects. | Not studied. |
| Gazdzinski et al., 2012 | Mouse, n = 35, F, 2.5-weeks-old | Gamma-rays; 0 or 7 Gy | Single  WBI | Whole brain | MRI (7T) | 6.5 weeks | A 2% to 8% reduction in brain volume (mainly in the CC, hippocampus, and cerebellum). | Reduced brain volume. | Not studied. |
| Zawaski et al., 2012 | Rat bearing C6-GFP expressing tumour, n = N/A, M, 6-to-8-weeks-old | Gamma-rays; 0 or 40 Gy (8 Gy/fraction/day) | Fractionated  PBI | Cortex | Histochemistry | 11 days | No significant cell death, but increased VEGF expression in peritumoral normal tissues. | Not studied. | Not studied. |
| Zhou et al., 2013 | Mouse, n = N/A, M, 6-to-8-weeks-old | Photons; 0 or 15 Gy (5 Gy/fraction) | Fractionated  WBI | Whole brain | RT-PCR, Histochemistry | 21 days | Increased VEGF and CXCL12 mRNA levels and expression compared to controls. | Not studied. | Not studied. |
| Blomstrand et al., 2014 | Rat, n = 15, M, 9-days (P9) and 6-months-old | Photons; 0 or 6 Gy | Single  WBI | Hippocampus | Cytochemistry, Histochemistry | 4 weeks | In P9 only, caspase-3 activity increased by 467% (up to 1 week), but  these animals had higher numbers of BrdU+ cells compared to adults and controls. | Not studied. | Not studied. |
| Cheng et al., 2014 | Rat, n = 96, M, 200-240 g | Gamma-rays; 0 or 60 Gy | Single  PBI | Cortex | Histochemistry, RT-PCR | 24 weeks | A significant upregulation of VEGF mRNA levels in irradiated cortex only. | Increased VEGF expression and mRNA levels in irradiated cortex. | Increased VEGF expression and mRNA levels up to 24 weeks post-IR. |
| Pius-Sadowska et al., 2016 | Mouse, n = 30, F, 6-to-8-weeks-old | Gamma-rays; 0 or 10 Gy | Single  WBI | Hippocampus, SVZ | Western blotting, Histochemistry | 7 days | Increased cell death  in SGZ. | Not studied. | Not studied. |
| Deng et al., 2017 | Mouse, n = 14, M, 8-week-old | X-rays; 0 or 10 Gy | Single  WBI | Cortex, White matter, Hippocampus | Histochemistry, Western blotting | 180 days | No tissue changes seen.  Increased expression of VEGF and Ang-2, but Ang-1 and Tie-2 expression levels were significantly decreased. | Cell swelling and chromatin condensation in cortex and white matter.  Increased expression of VEGF and Ang-2, but Ang-1 and Tie-2 levels were significantly decreased. | Increased expression of VEGF and Ang-2, but Ang-1 and Tie-2 levels were significantly decreased. |
| Allen et al., 2020 | Mouse, n = 24, F, 10-weeks-old | Conventional (0.09 Gy/s) or FLASH (6.9 x 10^6^ Gy/s) electron beams; 0 or 10 Gy | Single  WBI | Hippocampus, SVZ | Histochemistry | 1 month | Increased apoptosis in SVZ and DG, especially in conventional group. | No unspecific effects. | Not studied. |
