# Supplementary material for: A systematic review of normal tissue neurovascular unit damage following brain irradiation—Factors affecting damage severity and timing of effects
Source: Neurooncol Adv. 2024 Jun 13;6(1):vdae098. doi: 10.1093/noajnl/vdae098 (PMC11375288; doi:10.1093/noajnl/vdae098)
Supplement: vdae098_suppl_Supplementary_Table_S2 [file vdae098_suppl_supplementary_table_s2.docx]

**Supplementary table 2 | Evidence of radiation-induced neurovascular unit changes/damage in human studies**

| **Author, year** | **Subject model, Sex Age** | **IR type/ modality**  **& dose** | **Fraction,**  **brain volume** | **Brain region** | **Assay used** | **Follow-up time after IR** | **Acute effects: during to < a month post-IR** | **Delayed effects: from 1 to 6 months post-IR** | **Late effects: ≥ 6 months post-IR** |
| --- | --- | --- | --- | --- | --- | --- | --- | --- | --- |
| **Endothelial layer** | | | | | | | | | |
| *Vascular permeability* | | | | | | | | | |
| Qin et al., 1990 | Human patients with intra-cranial tumours, n = 14, sex/age; N/A | Photons; 0, or 30 to 40 Gy, (2 Gy/fraction/day, 5 times a week) | Fractionated  PBI | Whole brain | CT with 20 mCi Technetium-99m- glucoheptonate tracer | 8 months | A dose dependent increase in vessel permeability in the normal ipsilateral tissues compared to contralateral tissues. | Not studied. | At 8 months post-RT, vessel permeability in the normal irradiated tissue (n = 1) dropped from 214 (56% initial increase) to 134.5 (1.8% decrease) of the initial value (137). |
| Sharma et al., 2013 | Human brain ECs monolayers | Gamma-rays; 0, 2, 5, or 10 Gy | Single  Cell cultures | Whole brain | Cytochemistry, In vitro-photon microscopy, Impedance Spectroscopy for Trans-Endothelial Electrical Resistance (TEER) measurement | 1 day | A dose-dependent significant decrease in TEER values compared to those of controls.  No ovalbumin (70 kDa) leakage, but permeability of 10 kDa dextran tracer through the EC monolayers non-significantly increased.  Actin cytoskeleton, VE cadherin, and ZO-1 expression not affected, but PECAM-1 levels transiently reduced compared to those of controls. | Not studied. | Not studied. |
| *EC density and viability* | | | | | | | | | |
| Sharp et al., 2003 | Human brain ECs | Gamma-rays; 0 or 50 Gy | Single  Cell cultures | Whole brain | ELISA,  Cytochemistry | 3 days | No increased caspase-3 activity, but lactate dehydrogenase (LDH) activity significantly increased at 72 h post-IR compared to controls (p < 0.05). | Not studied. | Not studied. |
| *Surface and Junctional protein expression* | | | | | | | | | |
| Sharp et al., 2003 | Human brain ECs | Gamma-rays; 0 or 50 Gy | Single  Cell cultures | Whole brain | ELISA, Cytochemical staining | 3 days | Short-term upregulation of ICAM-1 (p < 0.001) (up to 1 day), and E-selectin (at 72 h) compared to controls (p < 0.05). | Not studied. | Not studied. |
| Sharma et al., 2013 | Human brain ECs monolayers | Gamma-rays; 0, 2, 5, or 10 Gy | Single  Cell cultures | Whole brain | Cytochemistry | 1 day | Actin cytoskeleton, VE cadherin, and ZO-1 expression not affected, but PECAM-1 levels transiently reduced compared to those of controls. | Not studied. | Not studied. |
| **Astrocytes** | | | | | | | | | |
| *Protein expression* | | | | | | | | | |
| Bylicky et al., 2019 | Human astrocyte cultures | X-rays; 0 or 10 Gy | Single  Cell cultures | Whole brain | Western blotting | 3 days | A significant increase in γ-H2AX foci in irradiated cells up to 48 h post-IR.  Up to 6 h post-IR, Ku70 protein levels increased by ~10-fold, and RAD51 and XRCC4 levels increased by >2-fold compared to control levels (p < 0.05). RPA levels significantly increased up to 2 h post-IR. | Not studied. | Not studied. |
| **Neurons** | | | | | | | | | |
| *Cell density and viability* | | | | | | | | | |
| Acharya et al., 2010 | Human neural stem cells | Gamma-rays; 0, 1, 2, or 5 Gy | Single  Cell cultures | Whole brain | Cytochemistry | 7 days | Increased number of undifferentiated neurons, and a reduction in neuronal cell numbers and metabolic ability (from day 3 to 5) compared to controls.  A ≥ 2-fold increase in caspase-3 signal from 6 h post-IR, reduced neural proliferation, cell cycle arrest for a day, increased γ-H2AX foci up to 1 h post-IR, and reduced levels of ROS/RNS and nitric oxide (by ≥ 3-fold), but superoxide levels were not affected. | Not studied. | Not studied. |
